# Structural basis of Ty1 integrase tethering to RNA polymerase III for targeted retrotransposon integration

**DOI:** 10.1038/s41467-023-37109-4

**Published:** 2023-03-28

**Authors:** Phong Quoc Nguyen, Sonia Huecas, Amna Asif-Laidin, Adrián Plaza-Pegueroles, Beatrice Capuzzi, Noé Palmic, Christine Conesa, Joël Acker, Juan Reguera, Pascale Lesage, Carlos Fernández-Tornero

**Affiliations:** 1 Centro de Investigaciones Biológicas Margarita Salas, CSIC, 28040 Madrid, Spain; 2 Aix-Marseille Université, CNRS, AFMB UMR 7257, 13288 Marseille, France; 3 Université Paris Cité, IRSL, Inserm, U944, CNRS, UMR7212, 75010 Paris, France; 4 Université Paris-Saclay, CEA, CNRS, Institute for Integrative Biology of the Cell (I2BC), 91198 Gif-sur-Yvette, France; 5 INSERM, AFMB UMR7257, 13288 Marseille, France

**Keywords:** Cryoelectron microscopy, Transcription, DNA recombination

## Abstract

The yeast Ty1 retrotransposon integrates upstream of genes transcribed by RNA polymerase III (Pol III). Specificity of integration is mediated by an interaction between the Ty1 integrase (IN1) and Pol III, currently uncharacterized at the atomic level. We report cryo-EM structures of Pol III in complex with IN1, revealing a 16-residue segment at the IN1 C-terminus that contacts Pol III subunits AC40 and AC19, an interaction that we validate by in vivo mutational analysis. Binding to IN1 associates with allosteric changes in Pol III that may affect its transcriptional activity. The C-terminal domain of subunit C11, involved in RNA cleavage, inserts into the Pol III funnel pore, providing evidence for a two-metal mechanism during RNA cleavage. Additionally, ordering next to C11 of an N-terminal portion from subunit C53 may explain the connection between these subunits during termination and reinitiation. Deletion of the C53 N-terminal region leads to reduced chromatin association of Pol III and IN1, and a major fall in Ty1 integration events. Our data support a model in which IN1 binding induces a Pol III configuration that may favor its retention on chromatin, thereby improving the likelihood of Ty1 integration.

## Abstract (web summary)

Cryo-EM structures of Ty1 integrase-Pol III complexes reveal determinants of Ty1 targeting upstream of Pol III-transcribed genes, and a functional impact of the integrase on Pol III activity that may increase the probability of Ty1 integration.

## Introduction

Most living organisms harbor transposable elements (TE) that, upon mobilization within the genome, participate in the adaptative response to environmental changes^1^. As mobile genetic elements, TEs also represent a potential threat for genome integrity and, accordingly, can cause human pathologies including cancer and aging^2–4^. To replicate and yet minimize genetic damage to its host, TEs have evolved the capacity of integrating into specific regions of the genome with minimal effects on cell function, generally through tethering to different cellular machineries that operate on the DNA^5^. Long-terminal repeat (LTR) retrotransposons constitute a group of TEs that, like retroviruses, replicate by reverse transcription of their mRNA in a double-stranded DNA (cDNA) that is subsequently integrated into the genome by their own integrase. Ty1 is the most active and abundant LTR-retrotransposon in *Saccharomyces cerevisiae*^6,7^. Ty1 preferentially integrates into nucleosomes within the first kilobase upstream of genes transcribed by RNA polymerase III (Pol III)^8,9^.

Pol III transcribes short genes encoding for untranslated RNAs such as transfer RNAs (tRNA), the 5S ribosomal RNA (rRNA) and the U6 spliceosomal RNA. Pol III is composed of 17 subunits that are organized into four architectural units^10–12^. Within the main unit, termed core, Pol III-specific subunits C160 and C128 form the DNA-binding cleft with the active site, while the assembly heterodimer AC40/AC19 is shared with RNA polymerase I (Pol I), and peripheral subunits Rpb5, Rpb6, Rpb8, Rpb10 and Rpb12 are shared with Pol I and RNA polymerase II (Pol II). The core is completed by subunit C11, involved in transcriptional pausing and RNA cleavage, termination and reinitiation^13–15^. Strong pausing induces enzyme backtracking associated with fraying of a few nucleotides from the RNA 3′ end, which, if not cleaved by the C11 C-terminal domain^13–15^, may lead to enzyme detachment from the DNA. Beside the core, the three remaining architectural units are formed by other Pol III-specific subunits: (i) a stalk including subunits C25 and C17, (ii) a nearby C82/C34/C31 heterotrimer sharing homology with transcription factor IIE (TFIIE), and (iii) a TFIIF-like heterodimer formed by subunits C37 and C53, which cooperate with C11 in termination and reinitiation^14^. Reinitiation is a unique Pol III property whereby the terminating enzyme can restart transcription on the same gene, assisted by initiation factors.

Genome-safe Ty1 integration upstream of Pol III-transcribed genes is mediated by an interaction between the Ty1 integrase (IN1) and the Pol III AC40 subunit^16,17^. Contact with additional Pol III subunits including C53, C34 and C31 has also been reported in vitro^18^. The IN1 N-terminal half retains the well-structured, phylogenetically-conserved oligomerization and catalytic domains of retroviral integrases, while the less-conserved C-terminal half is intrinsically disordered^19^. We recently showed that a stretch of residues next to the C-terminal end of IN1 is necessary and sufficient for association with AC40, IN1 recruitment to Pol III-transcribed genes and Pol III-mediated integration^16^. Nonetheless, the molecular details of this interaction have been obscured by lack of structural studies.

In this work, we present cryo-EM structures of yeast Pol III bound to IN1, in the absence and presence of a nucleic acid scaffold mimicking the transcription bubble. We also perform structure-based mutational analysis, both at residues involved in the interaction and at allosteric Pol III regions that show an altered configuration in the presence of IN1. Our results shed light on the mechanisms of Ty1 integration upstream of Pol III genes.

## Results

### Cryo-EM structures of Pol III bound to IN1

**Fig. 1:**
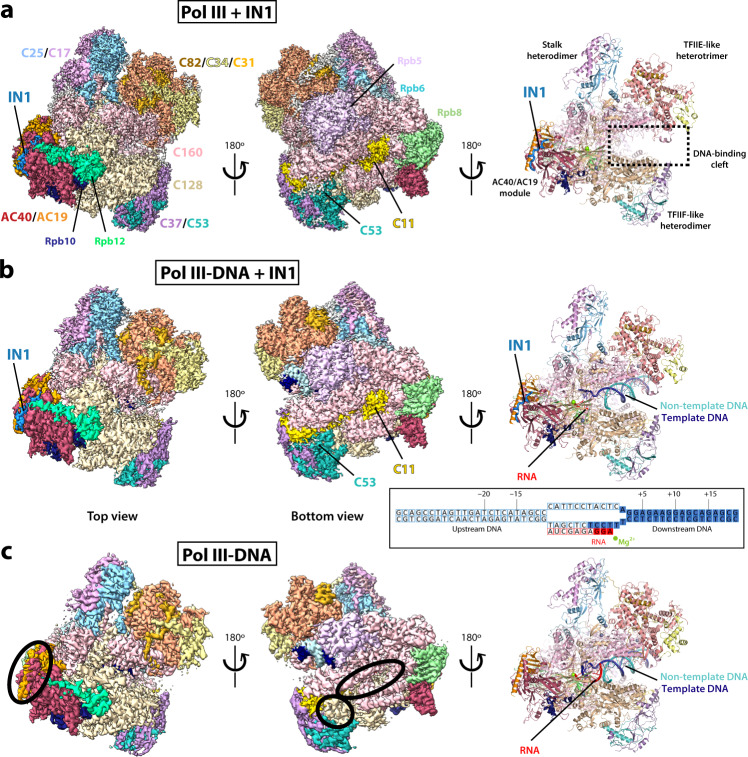
Overall structures of Pol III in the presence and absence of IN1. Cryo-EM maps (left and middle) and resulting structures (right) of Pol III bound to IN1 in the absence (**a**) or in the presence (**b**) of a mismatched transcription bubble (inset), and of Pol III complexed to the mismatched bubble in the absence of IN1 (**c**). All subunits and the main structural elements of Pol III are labeled. Mg^2+^ and Zn^2+^ ions are shown as green and gray spheres in the right panels. Additional densities attributed to IN1 (blue) or Pol III (yellow and cyan for C11 and C53, respectively) are indicated, while black ovals in panel ‘c’ indicate missing densities in the absence of IN1. Filled squares in the inset correspond to modeled nucleotides in the presence of IN1.

To gain structural insights into IN1 tethering on Pol III, we first formed an in vitro complex between recombinant IN1 produced in bacteria^19^ and the Pol III enzyme isolated directly from yeast^20^. The two species interacted in a 1:1 stoichiometry as shown by native gel electrophoresis (Supplementary Fig. 1a), demonstrating a direct interaction between the two enzymes. This interaction can be stabilized through mild crosslinking, despite the appearance of higher order oligomers that do not interfere with subsequent cryo-EM analysis. Using this sample, we reconstructed the cryo-EM structure of the Pol III bound to IN1 at 2.6 Å resolution (Fig. 1a; Supplementary Fig. 1b-d, map A; Supplementary Table 1). In parallel, we formed a Pol III complex with a transcription bubble mimetic comprising an 11-nucleotide mismatched DNA region, where one of the mismatched strands is hybridized to a 10-nucleotide RNA molecule (Fig. 1b, inset). Interestingly, IN1 is capable of interacting with this Pol III-DNA complex as with free Pol III (Supplementary Fig. 2a), suggesting that the interaction may occur at the periphery of this multi-protein enzyme. This preparation was employed to obtain the cryo-EM structure of the Pol III-DNA complex bound to IN1 at 3.1 Å resolution (Fig. 1b; Supplementary Fig. 2b–d, map D; Supplementary Table 1). Additionally, we obtained the cryo-EM structure of the Pol III-DNA complex in the absence of IN1 at 3.2 Å resolution (Fig. 1c; Supplementary Fig. 3, map E; Supplementary Table 1), which we used as a reference to assign map regions corresponding to IN1. Despite overall similarities (Supplementary Fig. 4a, b), the cryo-EM maps in the presence of IN1 exhibit remarkable differences respect to those obtained in the absence of IN1 (Fig. 1; Supplementary Fig. 4c), as described below.

**Fig. 2:**
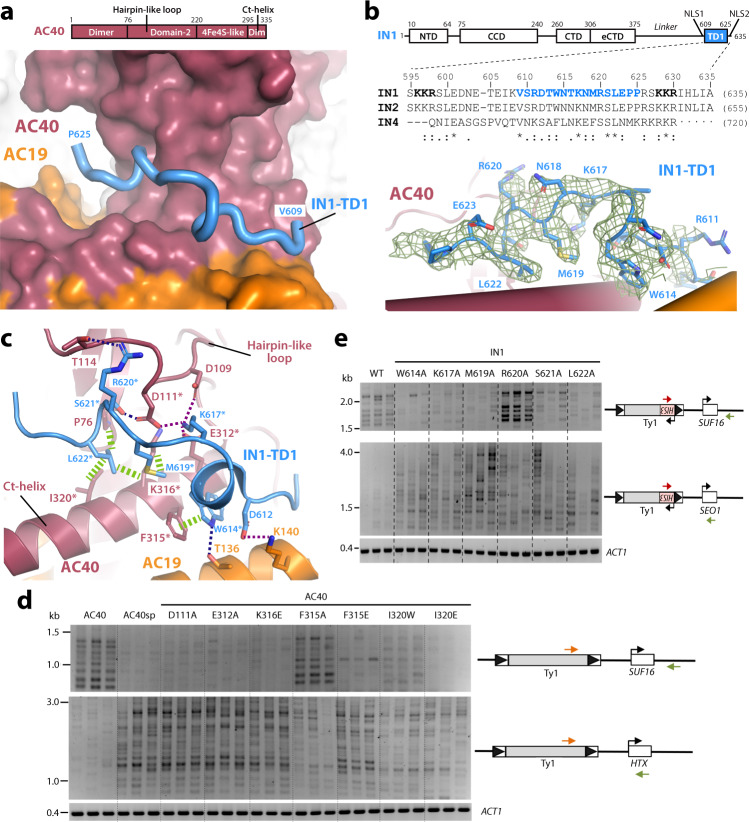
Interaction between AC40 and TD1. **a** Schematic representation of AC40 structural domains and close-up view of the Pol III bound to IN1 structure around AC40. Fully modeled TD1 is shown as a blue tube, while Pol III is shown as surface. **b** Schematic representation of IN1-TD1 including local alignment with Ty2 and Ty4 integrases (IN2 and IN4), with close-up view of the model (blue) and corresponding map (green mesh) around TD1. Hyphens and interpuncts in the IN4 sequence correspond to gaps and insertions, respectively. NTD, CCD, CTD and eCTD stand for N-terminal, catalytic core, C-terminal and extended C-terminal domains, respectively. NLS1 and NLS2 indicate nuclear localization signals, noted in bold font in the IN1 sequence. **c** Atomic details of the interaction between AC40 and IN1. H-bond, salt bridges and hydrophobic interactions are indicated with blue, purple and green dotted lines, respectively. Asterisks denote residues mutated in this study. **d** Detection of endogenous Ty1 insertions upstream of the *SUF16* Pol III-transcribed gene and the *HXT* subtelomeric genes (*HXT13*, *HXT15*, *HXT16* and *HXT17*) by PCR using primers indicated with orange and green arrows. Endogenous Ty1 retrotransposition was induced in cells growing at 20 °C during 3 days in YPD media. Total genomic DNA was extracted from three independent cultures. *ACT1* is genomic DNA quality control. **e** Detection of Ty1-*HIS3* de novo insertions generated in cells transformed by a plasmid expressing from the *GAL1* promoter WT or mutant Ty1-*his3AI* elements, bearing the indicated substitutions of conserved residues. Ty1-*HIS3* upstream of the *SUF16* Pol III-transcribed gene and the *SEO1* subtelomeric gene were detected by PCR using a primer in *HIS3* (red arrow) and a primer in the locus of interest (green arrow). Ty1 retrotransposition was induced by growing cells 5 days at 20 °C in the presence of galactose. Source data are provided as a Source Data file.

Maps in the presence of IN1 reveal a piece of elongated density at a crevice on the surface of subunit AC40, located between a hairpin-loop (residues 108–130) and the C-terminal helix of this subunit (Figs. 1, 2; Supplementary Fig. 5a). In the absence of IN1, this crevice harbors a molecule of detergent CHAPSO that we used to optimize cryo-EM grids for samples containing nucleic acids (Supplementary Fig. 5a), as reported^21^. We unambiguously assigned the elongated density from maps in the presence of IN1 to an IN1 segment (residues 609–625) proximal to its C-terminus (Fig. 2b). This segment contains the tethering motif of IN1 involved in Ty1 integration targeting (residues 617–622), previously identified through mutational analysis and referred to as targeting domain of Ty1 (TD1)^16^. Therefore, we hereafter use TD1 to designate the whole AC40-binding segment. TD1 corresponds to the sequence linking two nuclear localization signals (NLS1 and NLS2 in Fig. 2b, residues 596–598 and 628–630) that define a bipartite NLS^22,23^. Our reconstructions lack density attributable to either IN1 NLS, indicating that they are not conformationally constrained within the complex with Pol III and, therefore, may mediate simultaneous binding to importin-α in vivo. Moreover, conservation of TD1 in Ty2 and Ty4 retrotransposon integrases (Fig. 2b) suggests that an equivalent mechanism may operate for their interaction with AC40^17^ and preferential insertion upstream of Pol III-transcribed genes^7^.

Our maps in the presence of IN1 showed no density for the rest of IN1, including functional domains for Ty1 integration that locate at the N-terminal half of the protein (Fig. 2b, NTD and CCD). This is likely due to high flexibility of the IN1 linker region (residues 376–608), shown to exhibit intrinsic disorder^19^, that connects functional domains with TD1. Both in the presence and in the absence of IN1, our maps present an elongated density next to the AC40 hairpin-loop, also observed in a Pol III pre-termination complex^21^. We tentatively attribute this density to the N-terminus of the nearby subunit Rpb12 (Supplementary Fig. 5b). Residual density also appears next to the Pol III stalk in all maps, likely belonging to alternative conformations of a peripheral loop in subunit C17 (Supplementary Fig. 5c).

### Atomic details of the interaction between TD1 and AC40

In the structures of Pol III bound to IN1, TD1 adopts an extended conformation that includes a helical turn along the AC40 crevice and forms an intricate network of interactions with this subunit (Fig. 2b, c). Residues W614, M619 and L622 in IN1 establish hydrophobic contacts with P76, F315, I320 and the aliphatic chain of K316 in AC40. Additionally, R620 and S621 in IN1 establish one hydrogen bond (H-bond) each with D111 and T114 in AC40, respectively. Importantly, K617 in IN1 appears as a central residue coordinating three salt bridges with D109, D111 and E312 in AC40. The interaction between Pol III and TD1 is reinforced by contacts with subunit AC19, where residues T136 and K140 in this subunit establish an H-bond and a salt bridge with W614 and D612 in IN1, respectively. Our structural observations correlate remarkably well with reported mutational analysis of IN1, showing that individual mutations in residues K617, S621 or L622 disrupt the interaction with AC40 and alter Ty1 integration upstream of Pol III-transcribed genes^16^. This study also showed that mutation of residues N618 or E623, which in our structures point towards the solvent and are not conserved in Ty4 integrase (Fig. 2b), have no effect on the interaction between Pol III and IN1.

To investigate the role of individual AC40 residues in Ty1 integration, we produced single mutants at positions that appear critical according to our structures. As AC40 is essential, we checked that these mutations did not affect significantly cell growth or AC40 protein levels (Supplementary Fig. 6a, b). We previously showed that mutations altering the interaction between AC40 and IN1 do not affect Ty1 integration frequency but induce a redistribution of integration events into subtelomeric loci^16^. For all mutants, we observed a less than twofold decrease in retrotransposition frequency of a Ty1-*his3AI* reporter element expressed on plasmid from the *GAL1* promoter^24^ (Supplementary Fig. 6c), similar to that observed for the AC40 *Schizosaccharomyces pombe* ortholog (AC40sp), which behaves as a loss-of-interaction mutant^17^. In a qualitative PCR assay to monitor in vivo Ty1 insertion events (Fig. 2d), the *SUF16* tRNA gene was previously identified as a hotspot of Ty1 integration in the presence of wild-type AC40, while the *HXT* subtelomeric genes were preferred target sites when the interaction was compromised using AC40sp or Ty1 elements mutated in TD1^16,17^. In comparison to the wild-type protein, AC40 mutants D111A, E312A or K316E induced a significant reduction of Ty1 integration events upstream of *SUF16*, associated with a sharp increase in Ty1 integration at *HXT* loci, similar to the AC40sp mutant. An equivalent result was obtained when residue I320 in AC40 was mutated to either tryptophan or glutamate, suggesting that a bulky or charged residue at this position is sufficient to disrupt IN1 tethering to Pol III. Notably, I320W was designed because AC40sp contains a tryptophan at this position. Although to a lesser extent than I320E, I320W recapitulates the behavior of the AC40sp loss-of-function mutant. Additionally, mutation of F315 into alanine has no impact on Ty1 integration, while the F315E mutant behaves as the AC40sp mutant, highlighting the importance of a hydrophobic interaction between this residue and W614 in TD1. In accordance, the W614A mutant in IN1, lacking in our former IN1 mutational analysis^16^, impaired the two-hybrid interaction of TD1 with AC40 (Supplementary Fig. 7a, b). This mutation also exhibited a reduction in integration events upstream of *SUF16* and an increase in Ty1 integration at *HXT* loci as compared to wild-type IN1 (Fig. 2e), while it did not alter the overall Ty1 integration frequency (Supplementary Fig. 7c), indicating that this residue plays a major role in the interaction between Pol III and IN1. An equivalent effect is observed for the M619A mutant, as well as for previously-reported^16^ mutants K617A, S621A and L622A (Fig. 2e). While alanine substitution of R620 in IN1 impaired the interaction with AC40^16^, this mutation displayed an intermediate phenotype with increased integration at subtelomeres, as well as at *SUF16* (Fig. 2e), suggesting a more complex role of R620, which is not conserved in Ty4 integrase (Fig. 2b), in Ty1 integration targeting. We also prepared a double mutant of the AC19 subunit harboring the T136E and K140E changes, which do not affect cell growth (Supplementary Fig. 8a). Our PCR-based integration assay shows that the AC19 double mutant presents an intermediate integration profile that is similar to the wild-type upstream of *SUF16* but increased integration at subtelomeres (Supplementary Fig. 8b). This indicates some, but not essential, contribution of these AC19 residues to the interaction between Pol III and IN1.

We then tried to rescue the AC40sp loss-of-function mutant phenotype. Superposition of the AC40sp structure^25^ onto our structure of Pol III bound to IN1 shows that all residues of AC40 involved in TD1 binding are conserved with the exception of *S. cerevisiae* E312, which in *S. pombe* corresponds to V322 (Supplementary Fig. 8c, d). While conservative, changes from AC40sc residues F315 and I320 to AC40sp residues I325 and V330 could also affect the interaction with IN1. We produced the V322E mutation alone or combined with mutations I325F and V330I in the AC40sp loss-of-interaction mutant but did not observe recovery of Ty1 integration events at *SUF16*, with Ty1 insertions still occurring preferentially at subtelomeric loci in both mutants (Supplementary Fig. 8b). This indicates that differences beyond the TD1-binding crevice, possibly involving Pol III rearrangements, are relevant for Ty1 integration.

### The C11 C-terminal Zn-ribbon inserts into the Pol III funnel pore

**Fig. 3:**
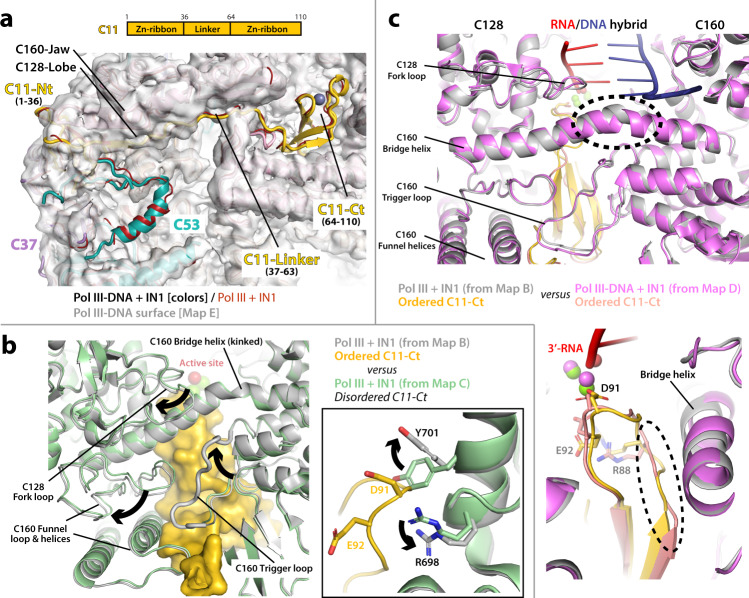
Structure of C11 in the presence of IN1. **a** Schematic representation of C11 and view around C11 in the structure of Pol III-DNA bound to IN1 (color ribbon) fitted in the Pol III-DNA map lacking IN1 (gray surface). Subunits C11, C37 and C53 are shown in yellow, purple and blue, respectively. The structure of Pol III bound to IN1 lacking DNA is shown in dark red, for comparison. **b** Structural comparison of the Pol III bound to IN1 models derived from focused classification using a mask around C11-Ct, showing presence (gray) and absence (green) of C11-Ct (yellow) in the Pol III funnel pore. Arrows indicate differences between the structures. The inset shows a close-up view around the C11 acidic loop and the gating tyrosine Y701 in subunit C128. **c** Structural superposition of Pol III bound to IN1 (gray) and Pol III-DNA bound to IN1 (magenta) structures, with close-up views around the bridge helix (upper panel) and C11-Ct (lower panel). Dotted circles mark a kink in the bridge helix and remodeling of the C11 acidic loop, respectively.

Besides a direct interaction between Pol III subunits AC40/AC19 and the TD1 region of IN1, binding of the latter associates with allosteric changes in the Pol III enzyme. A major change is observed in subunit C11, involved in RNA cleavage^13^, as well as in termination and reinitiation together with the C37/C53 heterodimer^14^. C11 comprises two Zn-ribbons (residues 1–36 and 64–110) connected by a flexible linker (Fig. 3a). In reported yeast Pol III structures^12,26^, which all lack IN1, the C-terminal Zn-ribbon (C11-Ct) is either disordered or occupies a peripheral location next to subunit Rpb5 (Supplementary Fig. 9a). Accordingly, our yeast Pol III-DNA map, which lacks IN1, shows no density for C11-Ct and half of the linker (Fig. 3a; Supplementary Fig. 3a). In contrast, our maps of Pol III bound to IN1 exhibit density for C11-Ct within the Pol III funnel pore, a region that allows access to the active site of the enzyme (Figs. 1 and 3a). An equivalent location for C11-Ct has only been observed in recent structures of human Pol III in complex with a transcription bubble^27–29^. Nevertheless, the human C11-Ct is retracted away from the Pol III active site by about 9 Å, compared to our structures of IN1-bound yeast Pol III (Supplementary Fig. 9b). Full insertion of C11-Ct inside the funnel pore is expected when RNA cleavage is required following enzyme backtracking, as shown for yeast TFIIS, the RNA cleavage factor in Pol II transcription^30^. This unanticipated location of yeast C11-Ct in our IN1-containing structures indicates that IN1 binding favors insertion of this domain within the funnel pore.

Focused 3D classification of the Pol III bound to IN1 dataset showed that approximately one third of the particles contain C11-Ct in the funnel pore (Supplementary Fig. 1, map B), while the remaining particles presented no density for this domain (Supplementary Fig. 1, map C). Comparison of structures derived from these two maps uncovered Pol III rearrangements for C11-Ct accommodation, mainly involving opening of the C160 funnel and C128 fork domains using a kink in the C160 bridge helix as hinge, associated with a fully ordered C160 trigger loop (Fig. 3b). This loop is disordered in reported apo or elongating yeast Pol III structures^12^. Additionally, residues R698 and Y701 in C128 switch their conformation to allow access of the C11 acidic loop, comprising residues 88-93 that are involved in RNA cleavage^31^, into the active site (Fig. 3b, inset). Strikingly, focused classification of the Pol III-DNA bound to IN1 dataset showed that virtually all particles contain C11-Ct in the pore (Fig. 1b; Supplementary Fig. 2, map D). In contrast, focused classification of the Pol III-DNA dataset, which lacks IN1, produced the opposite result (Fig. 1c; Supplementary Fig. 3, map E). In the structure of Pol III-DNA bound to IN1, the Pol III bridge helix presents a straight conformation, associated to a conformational change in the nearby C11 acidic loop, while the trigger loop is fully ordered and retracted (Fig. 3c). In yeast Pol II, an equivalent configuration with a straight bridge helix and a locked trigger loop has been observed in the reactivated intermediate of backtracking complexed with TFIIS^30^ (Supplementary Fig. 9c, left panel).

**Fig. 4:**
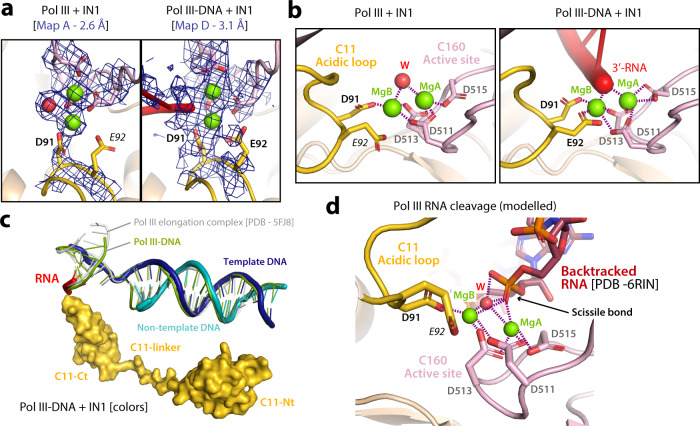
Pol III active site for RNA cleavage. **a** Active site model and map (blue mesh) in the structures of Pol III bound to IN1 (left panel) and Pol III-DNA bound to IN1 (right panel). **b** Close-up views of the active site in the structures of Pol III bound to IN1 (left panel) and Pol III-DNA bound to IN1 (right panel). Green and red spheres correspond to Mg^2+^ ions and water, respectively, while dotted lines indicate metal coordination. **c** Superposition between the structure of Pol III-DNA bound to IN1 (thick ribbon; RNA/DNA hybrid modelled from poor density, especially for RNA) and those of Pol III-DNA (green ribbon) and a canonical Pol III elongation complex (grey ribbon; PDB 5FJ8). The C11 subunit in Pol III-DNA bound to IN1 is shown as yellow surface, while the DNA template strand (TS) and non-template strand (NTS) are blue and cyan, respectively, and the RNA is red. **d** Model of a pre-catalytic complex for RNA cleavage using our Pol III bound to IN1 structure and an RNA molecule from the superposed structure of bacterial RNA polymerase in pre-catalytic state (PDB 6RIN).

The quality of our maps containing C11-Ct in the Pol III pore (maps B and D) enabled precise building of all residues and metal ions in the active site (Fig. 4a), where three catalytic aspartates (D511, D513, D515) from subunit C160 coordinate a Mg^2+^ ion (MgA in Fig. 4b). In addition, residue D91 in C11-Ct together with catalytic residues D511 and D513 coordinate a second Mg^2+^ ion (MgB in Fig. 4b) lying 3 Å away from MgA, thus creating a composite active site with two metals as postulated for Pol II^30,32^. Residue E92 in C11-Ct further coordinates MgB in the presence of nucleic acids, while its side chain is more flexible in their absence (Fig. 4b). Consistently, residues D91 and E92 in C11 are essential for RNA cleavage, while they are not involved in termination or reinitiation^13–15,33^. In the presence of nucleic acids poor density allows for fitting three base pairs of the RNA/DNA hybrid, in contrast with our Pol III-DNA structure or that reported for the yeast Pol III elongation complex^12^, where six and eight base pairs are respectively observed for the hybrid (Fig. 4c). This indicates that, in the presence of IN1, the interaction between Pol III and the hybrid is destabilized. Superposition with the structure of bacterial RNA polymerase in the pre-catalytic state^34^ allowed us to generate a model for RNA cleavage by Pol III (Fig. 4d). Altogether, these observations suggest that, in the presence of IN1, Pol III adopts a conformational state with C11-Ct in the pore that might influence Pol III function in vivo. An RNA extension assay shows a minor reduction of Pol III activity in the presence of IN1, especially for intermediate RNA products (Supplementary Fig. 10a), which may reflect reduced Pol III dissociation from pause sites. Moreover, a deletion mutant lacking C11-Ct (C11∆71-110; C11∆Ct) presents a twofold reduction in integration frequency and an altered profile of integration at *SUF16* relative to the wild-type (Supplementary Fig. 10c-d). In contrast to AC40 or IN1 loss-of-interaction mutants, we do not observe Ty1 integration at *HXT* subtelomeric loci, suggesting that IN1 remains bound to Pol III in the C11∆Ct mutant. As this strain is unable to grow at 20 °C (Supplementary Fig. 10b), which is optimal for retrotransposition, our assays were performed at 25 °C, where growth of this strain is partially affected and Ty1 overall retrotransposition still occurs but at suboptimal levels. This could mitigate the effect of C11∆Ct on Ty1 integration.

### The C11 N-terminal Zn-ribbon and linker associate with C37/C53

**Fig. 5:**
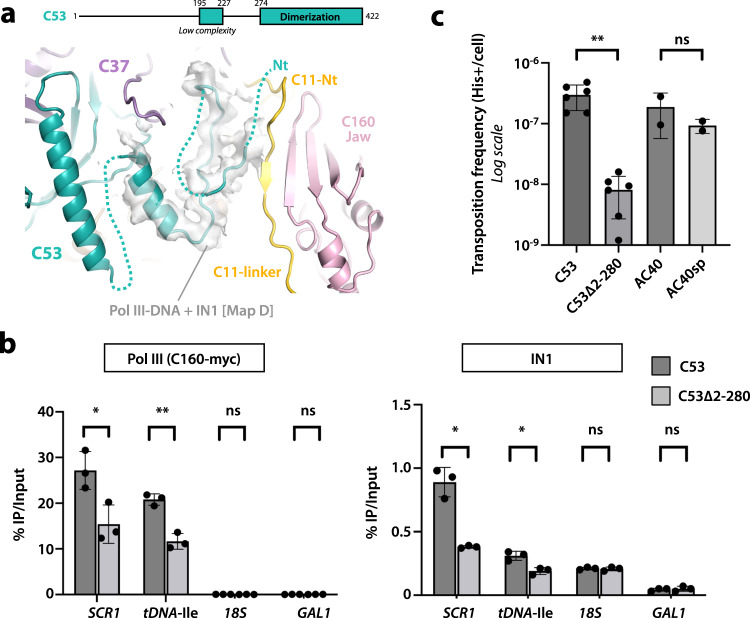
Role of C53-Nt in Ty1 integration. **a** Schematic representation of C53 and view of the Pol III-DNA bound to IN1 map around C53-Nt with the resulting structure. **b** Quantitative chromatin immunoprecipitation (ChIP) analysis of Pol III or IN1 enrichment at Pol III-transcribed genes. Immunoprecipitated DNA from WT or mutant *rpc53∆2-280* cells expressing a myc-tagged copy of C160 using anti-myc or anti-IN1 antibodies is expressed as a value relative to that of the input. The unique *SCR1* gene or the 16 genes of the *tDNA*-Ile family are representatives of Pol III-transcribed genes. The 18 S rDNA is transcribed by Pol I. *GAL1* ORF serves as a control. Values are mean ± SD, *n* = 3 experiments. *p*-values for C160-myc ChIP: *SCR1*, 0.0262; *tDNA-Ile*, 0.0025; *18S*, 0.3379*; GAL1*, >0.9999. *p*-values for IN1 ChIP: *SCR1*, 0.0161; *tDNA-Ile*, 0.0119; *18* *S*, 0.7700*; GAL1*, 0.6495. **c** Retrotransposition frequency of a chromosomal Ty1-*his3AI* reporter in WT and mutant *rpc53∆2-280* strains, induced at 25 °C. Values are mean ± SD, *n* = 3 experiments, each performed with four independent colonies. *p* value for C53∆2-280 vs. C53: 0.0032. *p* value for AC40sp vs. C53: 0.4899. For all panels, *p*-values are *p < 0.05; **p < 0.01; ns not significant. Two-sided Welch’s *t*-test was used. Source data are provided as a Source Data file.

In our structures, the C11 N-terminal Zn-ribbon (C11-Nt) is located between the C160 jaw, the C128 lobe and subunit C37 (Figs. 3a and 5a), in an equivalent position to that observed in our Pol III-DNA structure and reported structures in the absence of IN1^12^. We were able to model the entire C11 linker (residues 37-63) when C11-Ct is ordered (Supplementary Fig. 1, map B; Supplementary Fig. 2, map D), independent of the presence of nucleic acids. However, only the N-terminal third of the C11 linker (residues 37–47) is visible when the C11-Ct is disordered (Supplementary Fig. 1, map C; Supplementary Fig. 3, map E). In our structures, this segment forms a β-strand extending a four-stranded β-sheet in the C160 jaw domain (Fig. 5a). Only in the presence of IN1, ordering of the entire C11 linker associates with the nearby appearance of elongated densities that further extend the jaw β-sheet (Fig. 5a). These densities correspond to a portion of the C53 N-terminal region (C53-Nt, residues 1–274), which exhibits low-complexity^11^. Our maps in the presence of IN1 allowed modeling of an extended loop of C53 followed by an α-helix (residues 195–227) and a short strand, which become ordered next to the C11 linker. Ordering of C53 residues 195–227 in the same location has been observed in the Pol III pre-termination complex^21^ (Supplementary Fig. 11a), while in other reported Pol III structures the entire C53-Nt is disordered^12,26,35^. However, compared to the pre-termination complex, Pol III-DNA bound to IN1 exhibits an additional short strand and an open funnel that enables access of C11-Ct into the pore (Supplementary Fig. 11a). Interestingly, the C11-Nt and linker are essential for Pol III reinitiation^15^ and C11 acts concertedly with the C37/C53 heterodimer during termination and reinitiation^14^ and to prevent transcriptional arrest^33^, suggesting that Pol III might be more prone to terminate and/or reinitiate in the presence of IN1. Moreover, a potential role in Ty1 integration has been raised for the low-complexity region of subunit C53^18^.

To evaluate the biological relevance of this finding, we produced a C53 deletion mutant lacking the entire low-complexity, N-terminal region (C53∆2-280; C53∆Nt), reported previously^36^. Chromatin immunoprecipitation (ChIP) analysis shows that the C53-Nt truncation leads to decreased occupancy of both Pol III and IN1 at two representative Pol III-transcribed loci (Fig. 5b). Moreover, this mutant presents a growth defect that is more prominent at restrictive temperatures (Supplementary Fig. 11b), as well as a 30-fold decrease in integration frequency at 25 °C as compared to the wild-type strain (Fig. 5c). Such decrease is not observed in the presence of AC40sp loss-of-interaction mutant (Fig. 5c), indicating that C53 plays a role in Ty1 integration that is different from that of AC40. Consistently, the decrease in integration of C53∆Nt affects Ty1 insertions both upstream of Pol III-transcribed genes and at subtelomeric regions, in contrast to what is observed in the presence of AC40sp (Supplementary Fig. 11c), as reported^18^. Altogether, these results suggest that truncation of C53-Nt leads to a defect in Pol III association to chromatin while free Pol III remains bound to IN1, likely through subunits AC40/AC19, thus reducing Ty1 integration. This is in line with pull-down assays showing that, in this mutant, IN1 remains bound to Pol III despite lack of IN1 interaction with C37^18^.

### Partial ordering of C34 and C31 regions in the DNA-binding cleft

**Fig. 6:**
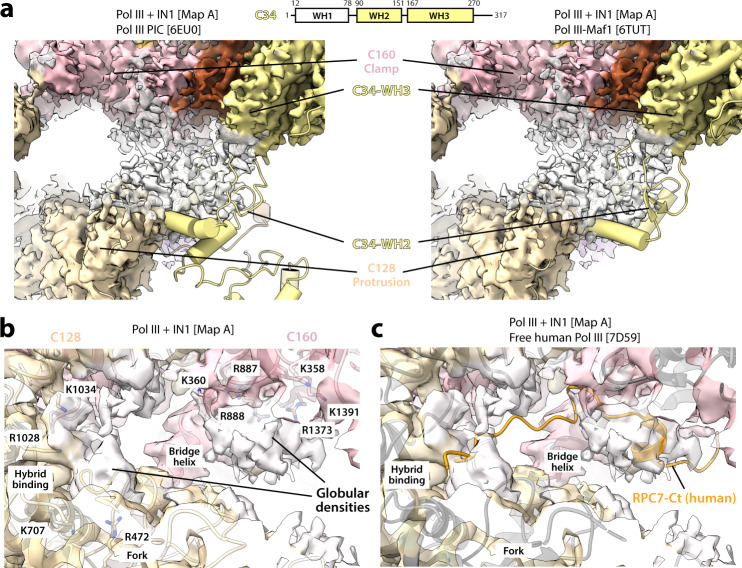
Partial ordering of C34 and C31 in the presence of IN1. **a** Schematic representation of C34 and view of the Pol III bound to IN1 map around C34, where the structure of Pol III from the reported Pol III PIC (PDB 6EU0; left) or Pol III-Maf1 complex (PDB 6TUT; right) has been fitted. View of the DNA-binding cleft in the Pol III bound to IN1 map with its corresponding model (**b**) or the structure of free human Pol III (PDB 7D59; **c**).

Subunit C34 contains three winged-helix (WH) domains connected by short linkers (Fig. 6a). The maps of Pol III bound to IN1 lacking nucleic acids (maps A–C) exhibit weak globular density at the rim of the DNA-binding cleft, between the C128 protrusion and the tip of the C160 clamp coiled-coil (Fig. 6a). The dimensions of this density and its position next to the C34-WH3 domain led us to hypothesize that it corresponds to the WH2 domain (residues 89–157) of this subunit. Consistently, C34-WH2 occupies an equivalent position in the structures of Pol III initiation intermediates^23–25^ or Pol III complexed to transcriptional repressor Maf1^37^ (Fig. 6a). Focused 3D classification confirmed the presence of this density in about 15% of the particles, also showing an additional density next to C34-WH2 that might account for the WH1 domain of this subunit (Supplementary Fig. 12a). Superposition of our structures with that of Pol III in the presence of TFIIIB^26,35,38^ shows that IN1 binding to Pol III is compatible with the formation of the pre-initiation complex (Supplementary Fig. 12b). As TFIIIB covers up to 60 base pairs upstream of Pol III-transcribed genes^39^, this is consistent with the absence of Ty1 integration within the first 80 base pairs upstream of tRNA genes^8,9^. These results could indicate that, in the presence of IN1, a fraction of the Pol III enzyme adopts a configuration that may favor interaction with TFIIIB, which would facilitate promoter recruitment or retention.

Besides, our maps of Pol III bound to IN1 lacking nucleic acids exhibit two globular pieces of density within the DNA-binding cleft. One locates next to the C160 bridge helix, while the other lies near the C128 fork and hybrid-binding domains (Fig. 6b). These densities, which are apparent but unassigned in reported structures of free yeast Pol III^12,26^, occupy the paths of downstream DNA and the RNA/DNA hybrid (Supplementary Fig. 12c). While the shape of these densities hampers model building, superposition with the structure of free human Pol III^29^ suggests that they may correspond to conserved regions of the C31 C-terminal tail (Fig. 6c). A third piece of elongated density occupying a depression in the RNA exit channel, and extending into the hybrid-binding region in the presence of IN1, could also correspond to a segment of C31 as shown for human Pol III^29^ (Supplementary Fig. 12d). Mutational analysis of the C31 C-terminal tail in yeast was either lethal or produced a strong growth defect, reflecting a role in preventing nucleic acid binding in free Pol III^29^. An equivalent role has been assigned to the DNA-mimicking loop of Pol I^40^, which occupies the cleft in the hibernating state of this enzyme^41,42^.

## Discussion

Targeted DNA integration is crucial to maintain genome integrity. The Ty1 retrotransposon takes advantage of the Pol III enzyme to integrate upstream of Pol III-transcribed genes, located in genome regions devoid of essential genes, thus protecting the yeast genome. The structures reported here provide evidence of primary IN1 binding through its TD1 on Pol III subunits AC40 and AC19 (Figs. 1 and 2). Our structural and mutational analyses allowed definition of the precise TD1 boundaries, comprising residues 609 to 625, and support that the TD1-AC40 interaction is sufficient for IN1 tethering on Pol III and for targeted integration (Fig. 2a, b).

Our maps show no density for the remaining of IN1, containing functional domains for integration of the Ty1 cDNA. This supports a scenario where the ~230 residue linker connecting the N-terminal half to the C-terminus of IN1 (Fig. 2b), which is disordered^19^, provides significant flexibility between the Pol III-tethering and the DNA-integrating modules of IN1. Ty1 integrates at DNA that is wrapped around the first to the third nucleosomes located upstream of Pol III-transcribed genes^8,9,17^ and, thus, a flexible linker is advantageous to reach varying distances while keeping strong tethering on genome-attached Pol III. This is in contrast with integrases from other LTR-retrotransposons or retroviruses, all lacking a large disordered linker connected to their respective targeting domains^5^. Retroviruses use transcriptional regulators of Pol II^43^, while the Ty3 integrase interacts with DNA-bound TFIIIB in a configuration that blocks Pol III binding, thus hampering transcription of the corresponding Pol III-transcribed gene^44^.

We show that IN1 binding associates with reordering of different Pol III regions that are distant from the IN1 binding site. Since IN1 binding to AC40 slightly alters the conformation of this subunit, this change is likely transmitted to other regions of Pol III (Supplementary Fig. 4c), thus producing an allosteric effect. Unexpectedly, in the presence of IN1, C11-Ct locates within the Pol III funnel pore, with its acidic loop complementing and remodeling the Pol III active site (Fig. 3). Notably, our structures show how a two-metal catalytic mechanism could operate for RNA cleavage, thus providing a model for this essential RNA polymerase activity (Fig. 4b, c). While this configuration may interfere with the transcription process, it may as well reduce Pol III pausing and its eventual detachment from DNA, as suggested by RNA extension assays (Supplementary Fig. 10a). This correlates with a mild reduction of integration frequency of a C11-Ct deletion mutant (Supplementary Fig. 10c, d). In addition, a portion of the low-complexity N-terminal region of subunit C53 orders next to the C11 linker (Fig. 5a), similar to a Pol III pre-termination complex^21^ (Supplementary Fig. 11a). Our mutational and ChIP analyses show that C53-Nt plays a role in Pol III binding to chromatin, which affects Ty1 integration while preserving the interaction between IN1 and subunit AC40. Altogether, we speculate that IN1 binding induces a Pol III configuration that increases its overall residence on the chromatin without significantly affecting the overall RNA production by this enzyme^45^. This is expected to enhance chances for IN1 to establish productive integration complexes at nucleosomes upstream of Pol III-transcribed genes and may, thereby, have contributed to the successful propagation of Ty1 in the *S. cerevisiae* genome.

Finally, lack of density for the two NLSs flanking TD1 could allow simultaneous binding of importin-α and Pol III by IN1^16^. Moreover, structural superposition with available structures shows no clash between IN1 and TFIIIB. These observations are consistent with the interaction between IN1 and Pol III taking place either in the nucleoplasm or while Pol III is bound on the DNA. Therefore, the TD1 motif not only provides strong binding but also increased likelihood for the tethering interaction to occur. This is especially relevant for the design of new gene therapy vectors able to target safe regions of the genome. The structures reported here represent a valuable tool in this respect, as they allow atomic-level understanding of the molecular mechanisms underlying targeted DNA integration upstream of genes transcribed by Pol III.

## Methods

### Growth media, yeast strains, and plasmids construction

*S. cerevisiae* strains used in this study were grown using standard methods and are listed in Supplementary Table 2. Plasmids were constructed using standard molecular biology procedures. Mutations were introduced in plasmids with Q5® Site-directed mutagenesis (NEB). All the constructs were validated by DNA sequencing (Eurofins Genomics). All plasmids and primers used in this study are reported in Supplementary Tables 3 and 4, respectively.

### Construction of mutant strains

To construct *RPC40* mutants, we used a *S. cerevisiae* yeast strain (LV1690) deleted for its *RPC40* genomic copy (AC40sc) and transformed with a centromeric plasmid bearing *Schizosaccharomyces pombe RPC40* gene (pTET-HA-AC40sp, *URA3*) to ensure cell viability^17^. Mutations were introduced in *RPC40* carried on a centromeric plasmid (pTET-HA-AC40sc, *TRP1*). Yeast strain was transformed with mutant pTET-HA-AC40sc plasmids and cells were plated on DO-TRP for three days at 30 °C. Isolated colonies were selected and spread on DO + 5-FOA media (5-fluoroorotic acid) to counter-select yeast cells with pTET-HA-AC40sp plasmid. Finally, sc*RPC40* mutant strains were validated by colony PCR, sequencing and western blots.

To construct V322E and V322E, I325F, V330I *spRPC40* mutants, the FYBL1-23D strain was transformed with pTET-HA-AC40sp plasmids harboring the mutations and cells were plated on DO-TRP for three days at 30 °C. Then, the genomic *RPC40* gene was deleted by gene replacement using an *rpc40::HphMX* deletion cassette. Finally, *spRPC40* mutant strains were validated by colony PCR and sequencing.

To construct T136E, K140E *RPC19* mutants, the LV33 strain was transformed with pTET-HA-AC19 WT or mutant centromeric plasmids and cells were plated on DO-TRP for three days at 30 °C. Then, the genomic *RPC19* gene was deleted by gene replacement using an *rpc19::NatMX* deletion cassette and the *RPC19* WT and mutant alleles were validated by colony PCR and sequencing.

To construct *RPC53* and *rpc53∆2-280* (C53∆Nt) strains, the JC3787 strain harboring a chromosomal Ty1-*his3AI* reporter element was transformed by pCW4 or pCW4mut centromeric plasmid expressing *RPC53* and *rpc53∆2-280*, respectively^36^, and selected on DO-LEU. The *RPC53* endogenous copy was then deleted using a *rpc53∆::KanMX* deletion cassette. The strains were checked by colony PCR and sequencing.

To construct the *rpc11(1-70)* (C11-∆Ct) mutant strain, the LV1454 strain was transformed by a centromeric plasmid harboring *RPC11* (pAMA171) or *rpc11∆1-70* (pAMA173) and the *RPC11* endogenous copy was subsequently deleted in the transformants by gene replacement using a *rpc11∆::KanMX* deletion cassette. The strains were checked by colony PCR and sequencing.

### Protein expression and purification

IN1 harboring a histidine tag for purification followed by a sso7d tag for solubility was produced as described^19^. *Escherichia coli* Rossetta(DE3) cells transformed with the IN1-expressing plasmid were grown in TB with ampicillin and chloramphenicol and grown at 25 °C to an OD600 equal to 0.6. Protein expression was induced by addition of 50 μM IPTG and the cells were grown overnight at 25 °C. Cells were harvested at 1100 g for 15 min at room temperature, washed with PBS 1× and weighed. Purification of IN1 was performed following described procedures^19^.

For Pol III, *S. cerevisiae* strain SC1613, encoding a tandem affinity-purification (TAP) tag at the C-terminus of subunit AC40, was provided by Cellzome AG (Heidelberg, Germany). Pol III was purified as described^20^ with some modifications. About 0.8 kg of cells were suspended in buffer A (250 mM Tris-HCl pH 7.4, 20% glycerol, 250 mM (NH_4_)_2_SO_4_, 1 mM EDTA, 10 mM MgCl_2_, 10 µM ZnCl_2_, 10 mM β-mercaptoethanol) and lysed at 4 °C with glass beads in a BeadBeater (BioSpec). After centrifugation, the supernatant was incubated overnight at 4 °C with 6 ml of IgG Sepharose (GE Healtcare), then the resin was washed with 20 column volumes of buffer B (50 mM Tris-HCl pH 7.4, 5% glycerol, 250 mM NaCl, 5 mM MgCl_2_, 10 µM ZnCl_2_, 5 mM DTT) and incubated overnight with tobacco etch virus (TEV) protease to elute the protein by removal of part of the TAP-tag. The TEV eluate was further purified using a Mono-Q (GE Healthcare) with a gradient from buffer B to the same buffer containing 1 M NaCl. Pol III eluted at ~360 mM NaCl. The protein was concentrated to 7-8 mg/ml, frozen with liquid nitrogen and stored at −80 °C until use.

### Preparation of Pol III complexes with IN1

For Pol III bound to IN1, Pol III and IN1 both at 0.5 µM were incubated in 20 mM HEPES-NaOH, pH 7.5, 170 mM NaCl, 2 mM DTT for 30 min at 4 °C. The complex was crosslinked following described procedures with minor modifications^42^. Briefly, the reaction was started by addition of 0.06%(v/v) of glutaraldehyde and, after 5 min incubation on ice, the remains of the cross-linking agent were quenched by adding 50 mM glycine and incubating 5 min on ice.

To prepare the mismatched transcription bubble, non-template DNA (5′-GCAGCCTAGTTGATCTCATAGCCCATTCCTACTCAGGAGAAGGAGCAGAGCG-3′), template DNA (5′-CGCTCTGCTCCTTCTCCTTTCCTCTCGATGGCTATGAGATCAACTAGGCTGC-3′) and RNA (5′-AUCGAGAGGA-3′) from Microsynth were incubated following described procedures^46^. The Pol III-DNA complex was assembled by incubating the transcription scaffold at equimolar amounts with the enzyme, at a final concentration of 0.4 μM, for 1 h at 20 °C in buffer E (10 mM Hepes pH 7.5, 150 mM NaCl, 5 mM MgCl_2_, 5 mM DTT). To obtain the Pol III-DNA complex bound to IN1, the sample was then mixed with 0.6 μM IN1 (final concentration) and incubated for 30 min at 20 °C in buffer E.

### Cryo-EM grid preparation and data acquisition

The crosslinked complex between Pol III and IN1 was concentrated to 1 µM, then 4 µl were applied to glow-discharged copper 300 mesh C-flat 1.2/1.3 holey carbon grids (Protochips) in the chamber of a FEI Vitrobot at 10 °C and 100% humidity. The grids were blotted for 3.5 s with blotting force −5 and vitrified by plunging into liquid ethane cooled with liquid nitrogen. Movies were acquired on a FEI Titan Krios (ThermoFisher) electron microscope at 300 keV using a K3 summit (Gatan) direct electron detector operated in ‘super-resolution’ mode at defocus values between –1.0 and –2.8 µm and a physical pixel size of 1.06 Å, using EPU software. A total of 3321 non-tilted movies and 8900 movies tilted by 20° with 40 frames each were collected with an accumulated total dose of 42.45 e-/Å^2^.

For Pol III-DNA in the presence or in the absence of IN1, 3 µl of the sample at 0.4 or 0.1 µM were supplemented with 8 mM CHAPSO (final concentration), applied to glow-discharged copper 300 mesh Quantifoil R1.2/1.3 grids coated with continuous carbon, and incubated in the chamber of a FEI Vitrobot at 24 °C and 100% humidity for 1 min. The grids were blotted for 4 sec with blotting force –5 and vitrified by plunging into liquid ethane cooled down to liquid nitrogen temperature. For Pol III-DNA bound to IN1, data were collected on a FEI Titan Krios electron microscope operated at 300 kV, using a K3 summit (Gatan) direct electron detector. Images were acquired at defocus values between −1.0 and −2.8 μm and a pixel size of 1.085 Å. A total of 9327 movies with 40 frames each were collected with an accumulated total dose of 45.12 e-/Å^2^. For Pol III-DNA in the absence of IN1, data were collected on a Talos Arctica electron microscope operated at 200 kV, using a Falcon III (FEI) direct electron detector in electron counting mode. Images were acquired at defocus values varying between −1.2 and −2.6 μm at a pixel size of 0.855 Å. A total of 2901 movies with 60 frames each were collected with an accumulated total dose of 30.96 e-/Å^2^.

### Cryo-EM data processing

For the Pol III bound to IN1, movies were aligned and dose-corrected using MotionCor2^47^ as implemented in Relion 3.0^48^. Global contrast transfer function (CTF) parameters were estimated using GCTF^49^. Around 1000 particles were picked manually to generate reference-free 2D classes that were used for template-based autopicking after low-pass filtering to 20 Å, followed by estimation of local defocus of the individual particles using GCTF. The remaining processing was performed in Relion 3.1 (Supplementary Fig. 1). Threefold binned particles were subjected to 3D classification and the best class with 885,560 particles was selected and subjected to another round of 3D classification. The five best classes with 643,858 particles in total were extracted with a 300 pixel box without binning and subsequently refined, followed by two rounds of CTF refinement^50^ and Bayesian polishing. Final post-processing was performed using automatic masking and B-factor sharpening, resulting in a map at 2.5 Å resolution. Five independent runs of 3D classification were performed using masks for the AC40 subunit, the stalk, the protrusion plus clamp head, C11-Nt and C11-Ct. For each case, the best classes were selected and refined. The AC40 mask yielded a map at 2.6 Å resolution with clear density for TD1, while the C11-Ct mask produced maps at 2.9 and 2.8 Å resolution where this domain is present and absent, respectively.

For Pol III-DNA bound to IN1, movies were aligned and dose-corrected using MotionCor2 as implemented in Relion 3.1, and their CTF parameters were estimated using CTFFIND4^51^. Approximately 550 particles were picked manually and reference-free 2D classes were generated, five of which were used for template-based autopicking after low-pass filtering to 20 Å. Approximately 2,437,000 particles were automatically selected and extracted with a 300 pixel box using Relion 3.1, also employed for subsequent processing (Supplementary Fig. 2). Three rounds of reference-free 2D classification yielded a stack of 469,620 good-quality particles that were refined using as reference the EMD-3180 map filtered to 60 Å. The resulting map was used as a reference for 3D classification to generate four classes. Two of these classes showing Pol III-like shape were joined together (305,253 particles total) and refined to a resolution of 3.8 Å. The resolution of the map was improved to 3.07 Å using particle polishing and CTF refinement.

For Pol III-DNA in the absence of IN1, movies were aligned and dose-corrected using patch motion correction and their CTF parameters were estimated using patch CTF estimation, both as implemented in cryoSPARC^52^. 667,000 particles were picked using blob picker with a minimum and maximum diameter of 160 and 210 Å, respectively. After inspection, 527,000 particles remained and were reference-free 2D classified. The final subset of 101,000 particles was refined using homogeneous 3D refinement and our Pol III-DNA bound to IN1 map, low-pass filtered to 60 Å, as starting model. Additional CTF and non-uniform refinements yielded a map with a resolution of 3.2 Å.

### Model building and refinement

The available structure of the Poll III bound to a transcription bubble (PDB code: 5FJ8) was fitted in the 3D maps using UCSF Chimera^53^ and employed as starting point for model building for Pol III subunits and the nucleic acid scaffold. A homology model of the C11 C-terminal Zn-ribbon was generated with Phyre2^54^ and used as reference for model building, while the C11 linker was manually modelled in Coot^55^. Available structures of Pol III complexed to melted DNA (PDB code: 6EU1) and the Pol III-Maf1 complex (PDB code: 6TUT) were used as reference to improve coordinates of the stalk C17/C25 heterodimer, the C82/C34/C31 heterotrimer and the C37/C53 heterodimer. The nucleic acid scaffold DNA model was adapted from a stalled Pol I elongation complex (PDB code: 6H67). Segments corresponding to IN1 and C53-Nt were manually-built de novo based only on our maps. The structures were refined using real-space refinement as implemented in Phenix^56^. Refinement statistics are summarized in Supplementary Table 1. Figures were prepared using PyMOL (Schrödinger Inc.) and UCSF Chimera^53^.

### RNA extension assay

The nucleic acid scaffold was prepared by mixing equal amounts of template DNA: 5′-CGTAGCGGTATCGTGGTCGAGCGTGTCCTGGTCTAG-3′, non-template DNA: 5′-CGCTCGACCACGATACCGCTACG-3′ and RNA: 5′-Alexa488-CGACCAGGAC-3′ in 20 mM Tris, pH 7.5, 150 mM KCl, heated to 95 °C and slow-cooled to 4 °C. For RNA elongation, 100 nM Pol III was pre-incubated, when required, with 200 nM IN1 in 20 mM Tris pH 7.5, 150 mM KCl, 5 µM ZnCl_2_, 10 mM DTT for 10 min at room temperature. Then 100 nM of the scaffold was added and incubated for another 10 min. To initiate the reaction, 1 mM of NTPs mix (Invitrogen) and 10 mM MgCl_2_ was added and incubated at 37 °C. The reaction was stopped by adding an equal amount of 2x RNA loading dye (8 M urea, 2× TBE, 0.02% bromophenol blue, 10% (v/v) glycerol) and heating to 95 °C for 5 min. The samples were loaded onto denaturing 20% polyacrylamide gel containing 7 M urea and visualized with a Fujifilm FLA-3000. For quantification, the percentage of each band respect to the total signal per lane was analyzed with the ImageJ-NIH software.

### Ty1-*his3AI* transposition assays

To estimate the frequency of retrotransposition of p*GAL1*-Ty1-*his3AI*, four independent transformants of each strain were grown to saturation for 2 days at 30 °C in liquid SC-URA containing 2% raffinose. Each culture was diluted thousand-fold in liquid SC-URA containing 2% galactose and grown for 5 days to saturation at 20 °C, which is the optimal temperature for Ty1 retrotransposition. Aliquots of cultures were plated on YPD (100 μl at 10^−5^) and SC-HIS (100 μl at 10^−2^). Plates were incubated for 3 days at 30 °C and colonies counted to determine the fraction of [HIS^+^] prototroph.

Similar experiments were performed in C53 and C11 mutant strains harboring a chromosomal Ty1-*his3AI* reporter, with the following differences. Four independent clones were grown at 30 °C to saturation in liquid YPD. Each culture was diluted in YPD, a thousand-fold for wild-type and C53 mutant strains and 250-fold for C11 mutant strains, and grown to saturation at 25 °C. Aliquots of cultures were plated on YPD (1 × 100 µl of a 1:20,000 dilution) and SC-HIS (2 × 2 ml). Plates were incubated for 4–5 days at 30 °C and colonies were counted to determine the fraction of [HIS+] prototrophs.

A retrotransposition frequency was calculated as the median of the ratios of number of [HIS+] cells to viable cells for each of the four independent clones. Retrotransposition frequencies were defined as the mean of at least three medians.

### PCR assays for detection of Ty1 integration events

To detect endogenous Ty1 insertions, three colonies of each yeast strain were inoculated overnight in YPD at 30 °C. The next day, each culture was diluted thousand-fold in YDP (or 250-fold for C11 mutants) to induce Ty1 retrotransposition for 3 days at 20 °C (or for 5 days at 25 °C for the experiments with C53 and C11 mutants) and total genomic DNA was extracted according to classical procedures^57^. For the detection of Ty1-*HIS3* insertions, p*GAL1*-Ty1-*his3AI* retrotransposition was induced as described in the previous section and total genomic DNA was extracted from yeast cultures grown at 20 °C for 5 days. Double-strand DNA (dsDNA) concentration was determined using Qubit™ fluorometric quantification (Thermo Fisher Scientific). Ty1-*HIS3* integrations upstream of *tG(GCC)C* (*SUF16*) were amplified with PCR primers O-AL27 and O-AB91 and at the *SEO1* subtelomeric gene with PCR primers O-AL27 and O-AL10. Endogenous Ty1 integrations upstream of *tG(GCC)C* (*SUF16*) were amplified with PCR primers O-AB46 and O-AB91 and at the *HXT* subtelomeric genes (*HXT13*, *HXT15*, *HXT16* and *HXT17*) with PCR primers O-AB46 and O-ABA27.

PCR reaction consisted of 30 ng of dsDNA (or 75 ng for detection of insertions at *HXT* or *SEO1*), 5 μl Buffer 5×, 0.5 μl dNTP 10 mM, 0.625 μl of each primer at 20 μM, 0,25 μl of Phusion DNA Polymerase (Thermo Scientific) in a 25 μl final volume. Amplification was performed with the following cycling conditions in ProFlex™ PCR System (Life Technologies) cycler: 98 °C 2 min, 30× [98 °C 10 s, 60 °C 30 s, 72 °C 1 min], 72 °C 5 min, and hold 4 °C. PCR products were separated on a 1.5% agarose gel.

### Chromatin immunoprecipitation

Chromatin immunoprecipitation (ChIP) was performed as previously described^16^ on crosslinked log-phase *RPC53* or *rpc53∆2-280* cells expressing a myc-tagged copy of C160, the largest subunit of Pol III. After chromatin purification and solubilization, DNA-protein complexes were immunoprecipitated using 25 µl of magnetic beads (Pan Mouse IgG or Protein A Dynabeads, Invitrogen) coated with anti-myc (1 µg, lab-made in mice from clone 9E10) or anti-IN1 antibodies (5 µl of lab-made serum produced in rabbits immunized with recombinant IN protein). The purified DNA samples were analyzed by quantitative real-time PCR using the SYBR Green PCR master Mix kit (Thermo Fisher) and an ABI PRISM 7500 (Applied Biosystems). The results were normalized with the input DNA PCR signals and indicated by relative IP in the graphs. Values are the average of three independent experiments.

### Statistical analysis

The two-sided Welch’s *t* test was used allowing unequal variance. *p < 0.05; **p < 0.01; ***p < 0.001; ns, not significant. Statistical tests were performed using GraphPad Prism version 9.0.0.

### Reporting summary

Further information on research design is available in the Nature Portfolio Reporting Summary linked to this article.

## Supplementary information

Supplementary Information

Reporting Summary

## Data Availability

The data that support this study are available from the corresponding authors upon request. Electron density maps and associated coordinates have been deposited in the Electron Microscopy and Protein Data Banks, respectively, as follows: Pol III + IN1 from focused classification on AC40 (EMD-14421/PDB 7Z0H), on C11-Ct with this domain in the funnel pore (EMD-14469/PDB 7Z30), and on C11-Ct with this domain disordered (EMD-14470/PDB 7Z31); Pol III-DNA + IN1 (EMD-14468/7Z2Z); and Pol III-DNA (EMD-16299/PDB 8BWS). Source data are provided with this paper.

## Source data

Source Data
