# Supplementary material for: Structural basis of Ty1 integrase tethering to RNA polymerase III for targeted retrotransposon integration
Source: Nat Commun. 2023 Mar 28;14:1729. doi: 10.1038/s41467-023-37109-4 (PMC10050235; doi:10.1038/s41467-023-37109-4)

Supplementary Data

Structural basis of Ty1 integrase tethering to RNA polymerase III for targeted retrotransposon integration

Phong Quoc Nguyen[1,2,$], Sonia Huecas[1,$], Amna Asif-Laidin[3,$], Adrián Plaza-Pegueroles[1], Beatrice Capuzzi[3], Noé Palmic[3], Christine Conesa[4] Joël Acker[4], Juan Reguera[2,5], Pascale Lesage[3*], Carlos Fernández-Tornero[1*]

[1]Centro de Investigaciones Biológicas Margarita Salas, CSIC, 28040 Madrid, Spain

[2]Aix-Marseille Université, CNRS, AFMB UMR 7257, 13288 Marseille, France

[3]Université Paris Cité, IRSL, Inserm, U944, CNRS, UMR7212, 75010 Paris, France

[4]Université Paris-Saclay, CEA, CNRS, Institute for Integrative Biology of the Cell (I2BC), 91198 Gif-sur-Yvette, France

[5]INSERM, AFMB UMR7257, 13288 Marseille, France

[$]Equal contribution

[*]Corresponding authors: cftornero@cib.csic.es; pascale.lesage@inserm.fr

This PDF file includes:

Supplementary Tables 1 to 4

Supplementary Figs. 1 to 12

Source Data for Supplementary Figs. 1, 2, 6 to 8, 10 and 11

Supplementary Table 1. Cryo-EM data collection and refinement statistics

	**Pol III + IN1**			**Pol III-DNA + IN1**	**Pol III-DNA**
Sample support	C-flat 1.2/1.3<br>300 mesh<br>Holey carbon			Quantifoil 1.2/1.3<br>300 mesh<br>Continuous carbon	Quantifoil 1.2/1.3<br>300 mesh<br>Continuous carbon
Microscope	Titan Krios			Titan Krios	Talos Arctica
Detector	K3			K3	Falcon III
Voltage (kv)	300			300	200
Number of Frames	40			40	60
Dose (e^-/Å^2)	42.45			45	31
Exposure time (s)	3			3	3
Pixel size (Å/pixel)	1.06			1.085	0.855
Number of grids	1			1	1
Days of data collection	3			2	3
Collected micrographs	12221			9327	2901
Particles after 2D class	Not applicable			469620	101928
Particles after 3D class	885560			Not applicable	Not applicable
Map name	Map A	Map B	Map C	Map D	Map E
Final number of particles	226817	143021	273119	305253	101928
Resolution (Å)	2.65	2.92	2.76	3.07	3.23
Accuracy Rotations (°)	0.597	0.733	0.714	0.631	Not applicable
AccuracyTranslations (pixel)	0.322	0.507	0.466	0.446	Not applicable
Sharpening B-factor (Å^2)	-49.3	-62.1	-60.2	-73.5	-92.5
Ramachandran plot					
Outliers (%)	0.00	0.00	0.00	0.00	0.02
Allowed (%)	7.17	7.04	5.13	7.60	7.54
Favoured (%)	93.83	92.96	94.27	92.40	92.44
Map CC (around atoms)	0.76	0.77	0.76	0.76	0.80
RMSD bond lengths (Å)	0.005	0.005	0.003	0.003	0.004
RMSD bond angles (°)	0.775	0.749	0.651	0.625	0.594
All-atom clashscore	12.49	12.83	10.87	9.09	10.39
Rotamer outliers (%)	0.93	0.74	0.61	0.46	0.23
C-beta deviations	0.04	0.02	0.00	0.06	0.00
FSC(model-map) = 0.5	2.85	2.81	2.79	3.26	3.5
EMDB code	14421	14469	14470	14468	16299
PDB code	7Z0H	7Z30	7Z31	7Z2Z	8BWS

Supplementary Table 2. Yeast Strains used in this study

Strain code	Name	Genotype	Origin	Purpose
LV174	*spt3-101 rad52Δ*	*MATα ura3Δ851 trp1Δ63 his3Δ200 spt3-101 Δrad52::TRP1*	Ref. 16	Supp. Fig. 7
PJ69-4A	*Two-hybrid strain*	*MATα trp1-901 leu2-3,112 ura3-52 his3Δ200 gal4Δ gal80Δ LYS2::GAL1-HIS3 GAL2-ADE2 met2::GAL7-lacZ*	James et al., 1996[1]	Supp. Fig. 7
FYBL1-23D	*WT*	*MATα ura3Δ851 trp1Δ63 his3Δ200*	Morillon et al., 2000[2]	Mat & Meth.
LV1635	*RPC40sc (AC40sc)*	*MATα ura3Δ851 trp1Δ63 his3Δ200 rpc40Δ::HphMx + pTET-HA-RPC40Sc (TRP1)*	This study	Fig. 2, Supp. Fig. 6
LV1636	*RPC40sp (AC40sp)*	*MATα ura3Δ851 trp1Δ63 his3Δ200 rpc40Δ::HphMx + pTET-HA-RPC40Sp (TRP1)*	This study	Fig. 2, Supp. Fig. 6
LV1690	*RPC40sp (AC40sp)*	*MATα ura3Δ851 trp1Δ63 his3Δ200 rpc40Δ::HphMx + pTET-HA-RPC40Sp (URA3)*	This study	Fig. 2, Supp. Fig. 6
LV1857	*RPC40sc (AC40sc) F315A*	*MATα ura3Δ851 trp1Δ63 his3Δ200 rpc40Δ::HphMx + pTET-HA-RPC40Sc* $F_{315}A$ *(TRP1)*	This study	Fig. 2, Supp. Fig. 6
LV1859	*RPC40sc (AC40sc) F315E*	*MATα ura3Δ851 trp1Δ63 his3Δ200 rpc40Δ::HphMx + pTET-HA-RPC40Sc* $F_{315}E$ *(TRP1)*	This study	Fig. 2, Supp. Fig. 6
LV1863	*RPC40sc (AC40sc) D111A*	*MATα ura3Δ851 trp1Δ63 his3Δ200 rpc40Δ::HphMx + pTET-HA-RPC40Sc* $D_{111}A$ *(TRP1)*	This study	Fig. 2, Supp. Fig. 6
LV1873	*RPC40sc (AC40sc) E312A*	*MATα ura3Δ851 trp1Δ63 his3Δ200 rpc40Δ::HphMx + pTET-HA-RPC40Sc* $E_{312}A$ *(TRP1)*	This study	Fig. 2, Supp. Fig. 6
LV1869	*RPC40sc (AC40sc) I320E*	*MATα ura3Δ851 trp1Δ63 his3Δ200 rpc40Δ::HphMx + pTET-HA-RPC40Sc* $I_{320}E$ *(TRP1)*	This study	Fig. 2, Supp. Fig. 6
LV1871	*RPC40sc (AC40sc) I320W*	*MATα ura3Δ851 trp1Δ63 his3Δ200 rpc40Δ::HphMx + pTET-HA-RPC40Sc* $I_{320}W$ *(TRP1)*	This study	Fig. 2, Supp. Fig. 6
LV1897	*RPC40sc (AC40sc) K316E*	*MATα ura3Δ851 trp1Δ63 his3Δ200 rpc40Δ::HphMx + pTET-HA-RPC40Sc* $K_{316}E$ *(TRP1)*	This study	Fig. 2, Supp. Fig. 6
JC3787	Ty1-*his3AI*-3114	*MATα his3Δ1 leu2Δ0 lys2Δ0 ura3Δ0 Ty1-his3AI[Δ1]-3114*	Mou et al., 2006[3]	Mat & Meth.
YJR29	Ty1-*his3AI*-3114 *RPC40sp*	*MATα his3Δ1 leu2Δ0 lys2Δ0 ura3Δ0 Ty1-his3AI[Δ1]-3114 rpc40Δ::HphMx + pTET-HA-RPC40sp (TRP1)*	This study	Supp. Fig. 10
YJR30	Ty1-*his3AI*-3114 *RPC40sc*	*MATα his3Δ1 leu2Δ0 lys2Δ0 ura3Δ0 Ty1-his3AI[Δ1]-3114 rpc40Δ::HphMx + pTET-HA-RPC40sc (TRP1)*	This study	Supp. Fig. 10
LV1946	*RPC40sp (AC40sp) V322E*	*MATα ura3Δ851 trp1Δ63 his3Δ200 rpc40Δ::HphMx + pTET-HA-RPC40Sp* $V_{322}E$ *(TRP1)*	This study	Supp. Fig. 8

LV1948	*RPC40sp (AC40sp) V322E, I325F, V330I*	*MATα ura3Δ851 trp1Δ63 his3Δ200 rpc40Δ::HphMx + pTET-HA-RPC40Sp* $V_{322}E$ $I_{325}F$ $V_{330}I$ *(TRP1)*	This study	Supp. Fig. 8
LV1950	*RPC19 (AC19)*	*MATα ura3Δ851 trp1Δ63 his3Δ200 rpc19Δ::NatMx + pTET-HA-RPC19*	This study	Supp. Fig. 8
LV1953	*RPC19 (AC19) T136E, T140E*	*MATα ura3Δ851 trp1Δ63 his3Δ200 rpc19Δ::NatMx + pTET-HA-RPC19* $T_{136}E$, $_{T140}E$	This study	Supp. Fig. 8
LV1815	Ty1-*his3AI*-3114 *RPC53*	*MATα his3Δ1 leu2Δ0 lys2Δ0 ura3Δ0 Ty1-his3AI[Δ1]-3114*	This study	Fig. 5, Supp. Fig. 11
LV1817	Ty1-*his3AI*-3114 *rpc53Δ2-280*	*MATα his3Δ1 leu2Δ0 lys2Δ0 ura3Δ0 Ty1-his3AI[Δ1]-3114* *rpc53Δ::KanMX + prpc53Δ2-280 (LEU2)*	This study	Fig. 5, Supp. Fig. 11
LV1954	Ty1-*his3AI*-3114	*MATα his3Δ1 leu2Δ0 lys2Δ0 ura3Δ0 trp1Δ:: NatMX Ty1-his3AI[Δ1]-3114*	This study	Mat & Meth.
LV1938	Ty1-*his3AI*-3114 *RPC160-MYC* *RPC53*	*MATα his3Δ1 leu2Δ0 lys2Δ0 ura3Δ0 trp1Δ:: NatMX Ty1-his3AI[Δ1]-3114* *RPC160*-myc-Kl*URA3* *rpc53::KanMx + pRPC53 (LEU2)*	This study	Fig. 5
LV1940	Ty1-*his3AI*-3114 *RPC160-MYC* *rpc53Δ2-280*	*MATα his3Δ1 leu2Δ0 lys2Δ0 ura3Δ0 trp1Δ:: NatMX Ty1-his3AI[Δ1]-3114* *RPC160*-myc-Kl*URA3* *rpc53::KanMx + prpc53Δ2-280 (LEU2)*	This study	Fig. 5
LV1955	Ty1-*his3AI*-3114 *RPC11*	*MATα his3Δ1 leu2Δ0 lys2Δ0 ura3Δ0 trp1Δ::NatMX Ty1-his3AI[Δ1]-3114* *rpc11Δ::KanMX* + p*RPC11*(*URA3*)	This study	Supp. Fig. 8
LV1959	Ty1-*his3AI*-3114 *RPC11(1-70)*	*MATα his3Δ1 leu2Δ0 lys2Δ0 ura3Δ0 trp1Δ::NatMX Ty1-his3AI[Δ1]-3114* *rpc11Δ::KanMX* + p*RPC11*(1-70) (*LEU2*)	This study	Supp. Fig. 8

(1) James, P., Halladay, J. & Craig, E. A. Genomic libraries and a host strain designed for highly efficient two-hybrid selection in yeast. *Genetics* **144**, 1425–1436 (1996).

(2) Morillon, A., Springer, M. & Lesage, P. Activation of the Kss1 invasive-filamentous growth pathway induces Ty1 transcription and retrotransposition in *Saccharomyces cerevisiae*. *Mol. Cell Biol.* **20**, 5766–5776 (2000).

(3) Mou, Z., Kenny, A. E. & Curcio, M. J. Hos2 and Set3 promote integration of Ty1 retrotransposons at tRNA genes *Saccharomyces cerevisiae*. *Genetics* **172**, 2157–2167 (2006).

Supplementary Table 3. Plasmids used in this study

Name	Vector	Genotype	Origin	Purpose
pAT36	pAS2ΔΔ	2μ *AmpR TRP1 GBD-AC40*	Ref. 17	Supp. Fig. 7
pAL1	pACTII	2μ *AmpR LEU2 GAD-IN5*$_{\Delta TD+bNLS}$	Ref. 16	Supp. Fig. 7
pAL10	pACTII	2μ *AmpR LEU2 GAD-IN5*$_{\Delta TD+bNLS}$ *K*$_{617}$*A*	Ref. 16	Supp. Fig. 7
pNP67	pACTII	2μ *AmpR LEU2 GAD-IN5*$_{\Delta TD+bNLS}$ *W*$_{614}$*A*	This study	Supp. Fig. 7
pPL2	p*GAL1*-Ty1-*his3AI*	2μ *AmpR URA3 GAL1p*-Ty1-*his3AI*	Ref. 24	Fig. 2, Supp. Fig. 7
pCG1	p*GAL1*-Ty1-*his3AI*	2μ *AmpR URA3 GAL1p*- Ty1-*his3AI* (IN1-K$_{617}$A)	Ref. 16	Fig. 2, Supp. Fig. 7
pCG2	p*GAL1*-Ty1-*his3AI*	2μ *AmpR URA3 GAL1p*- Ty1-*his3AI* (IN1-M$_{619}$A)	Ref. 16	Fig. 2, Supp. Fig. 7
pCG3	p*GAL1*-Ty1-*his3AI*	2μ AmpR *URA3 GAL1p*- Ty1-*his3AI* (IN1-R$_{620}$A)	Ref. 16	Fig. 2, Supp. Fig. 7
pCG4	p*GAL1*-Ty1-*his3AI*	2μ *AmpR URA3 GAL1p*- Ty1-*his3AI* (IN1-S$_{621}$A)	Ref. 16	Fig. 2, Supp. Fig. 7
pCG5	p*GAL1*-Ty1-*his3AI*	2μ *AmpR URA3 GAL1p*-Ty1-*his3AI* (IN1-L$_{622}$A)	Ref. 16	Fig. 2, Supp. Fig. 7
pNP71	p*GAL1*-Ty1-*his3AI*	2μ *AmpR URA3 GAL1p*- Ty1-*his3AI* (IN1-W$_{614}$A)	This study	Fig. 2, Supp. Fig. 7
pRM2	pCM185	CEN *AmpR TRP1 pTET-HA-RPC40*	Ref. 17	Fig. 2, Supp. Fig. 6
pRM3	pCM185	*CEN AmpR TRP1 pTET-HA-RPC40Sp*	Ref. 17	Fig. 2, Supp. Fig. 6
pAT12	pCM189	*CEN AmpR URA3 pTET-HA-RPC40Sc*	Ref. 17	Fig. 2, Supp. Fig. 6
pAT30	pCM189	*CEN AmpR URA3 pTET-HA-RPC40Sp*	Ref. 17	Fig. 2, Supp. Fig. 6
pNP52	pCM185	CEN *AmpR TRP1 pTET-HA-RPC40 D*$_{111}$*A*	This study	Fig. 2, Supp. Fig. 6
pNP54	pCM185	CEN *AmpR TRP1 pTET-HA-RPC40 E*$_{312}$*A*	This study	Fig. 2, Supp. Fig. 6
pNP57	pCM185	CEN *AmpR TRP1 pTET-HA-RPC40 F*$_{315}$*A*	This study	Fig. 2, Supp. Fig. 6
pNP58	pCM185	CEN *AmpR TRP1 pTET-HA-RPC40 F*$_{315}$*E*	This study	Fig. 2, Supp. Fig. 6
pNP60	pCM185	CEN *AmpR TRP1 pTET-HA-RPC40 I*$_{320}$*E*	This study	Fig. 2, Supp. Fig. 6
pNP61	pCM185	CEN *AmpR TRP1 pTET-HA-RPC40 I*$_{320}$*W*	This study	Fig. 2, Supp. Fig. 6
pNP69	pCM185	CEN *AmpR TRP1 pTET-HA-RPC40 K*$_{316}$*E*	This study	Fig. 2, Supp. Fig. 6
pNP83	pCM185	CEN *AmpR TRP1 pTET-HA-RPC19*	This study	Supp. Fig. 8
pNP85	pCM185	CEN *AmpR TRP1 pTET-HA-RPC19 T*$_{136}$*E, K*$_{140}$*E*	This study	Supp. Fig. 8
pNP78	pCM185	CEN *AmpR TRP1 pTET-HA-RPC40sp V*$_{322}$*E*	This study	Supp. Fig. 8
pNP86	pCM185	CEN *AmpR TRP1 pTET-HA-RPC40sp V*$_{322}$*E, I*$_{325}$*F, V*$_{330}$*I*	This study	Supp. Fig. 8
pCW4	pRS315	CEN *AmpR LEU2 RPC53-V5*	Ref. 36	Fig. 5, Supp. Fig. 11
pCW4mut	pRS315	CEN *AmpR LEU2 rpc53Δ2-280-V5*	Ref. 36	Fig. 5, Supp. Fig. 11
pAMA171	pRS316	*CEN AmpR URA3 RPC11*	This study	Supp. Fig. 10
pAMA173	pRS316	*CEN AmpR ura3:LEU2 RPC11(1-70)*	This study	Supp. Fig. 10

Supplementary Table 4. Primers used in this study

Primer	Sequence 5'- 3'	References
Site-directed mutagenesis		
O-AL112_IN1W614A Fw	AAATACTAAGAATATGCGTAGTTTAG	This study
O-AL113_IN1W614A Rev	GCTGTGTCTCGTGATACCTTAATTTC	This study
O-AL114_AC40sc D111A Fw	AATGCTCACATGGGTTGATAGTAATTTG	This study
O-AL115_AC40sc D111A Rev	GCAGGGTCAACTTTTAATGGAACCAAG	This study
O-AL141_AC40sc E312A Fw	GACACCAGAAGCGATTTTTTTCAAATCCGTCAG	This study
O-AL143_AC40sc E312A Rev	ATGGCACCAGCGCTTTCT	This study
O-AL123_AC40sc F315A Fw	TAAATCCGTCAGGATTTTAAAG	This study
O-AL124_AC40sc F3125 Rev	GCAAAAATTTCTTCTGGTGTCATG	This study
O-AL125_AC40sc F315I Fw	CAAATCCGTCAGGATTTTAAAG	This study
O-AL134_AC40sc F315I Rev	ATAAAAATTTCTTCTGGTGTCATG	This study
O-AL130_AC40sc I320W Fw	GTTAAAGAATAAGGCTGAGTATTTG	This study
O-AL131_AC40sc I320W Rev	CACCTGACGGATTTGAAAAAAATTTC	This study
O-AL132_AC40sc I320E Fw	ATTAAAGAATAAGGCTGAGTATTTG	This study
O-AL133_AC40sc I320E Rev	TCCCTGACGGATTTGAAAAAAATTTC	This study
O-AL169_AC40sc K316E Fw	GTTGAAGGCAGAATGCATTTTAG	This study
O-AL170_AC40sc K316E Rev	GAAATTTCTTGACCAGGC	This study
O-AL173_AC40sp V322E Fw	GAAGCCCGATGAATTGTTCATTAAAAG	This study
O-AL174_AC40sp V322E Rev	ATAATACCGGTGCTTTCAAC	This study
O-NP78_AC40sp I325F+V330I Fw	ATTGCAATCCTAAAATCGAAATGTCTAGCCG	This study
O-NP79_AC40sp I325F+V330I Rev	GCTTTTAAAGAACAATTCATCGGGCTTCATAAT AC	This study
O-NP73_AC19sc Fw	GGGGGATCCATGTATCCGTATGATGTGCCGGA TTATGCGACTGAAGACATCGAACAAAA	This study
O-NP74_AC19sc Rev	CTCCTGCAGGGACTACATGCTCTTGATTTTTTC AGT	This study
O-NP77_AC19sc T136E +K140E Fw	CTCCTGCAGGGACTACATGCTCTCGATTTTTTC CTCAAACTTAGA	This study
PCR selectivity		
O-AB46_TYB-Out 2	GTGATGACAAAACCTCTTCCG	Dakshinamurthy et al., 2010[1]

O-AB91	TTTTAGAGTGACACCATCGTAC	Dakshinamurthy et al., 2010[(1)]
O-ABA27_HXT13,15,16,17	GACATGGGCCCCTGTTGCTTATATTGT	Ref. 18
O-AL10_SEO1	CGTTGGTGCTGCATATGTCA	Ref. 16
O-AL27-HIS3	GCAAGAGAGATCTCCTACTTTC	Ref. 16
Chromatin immunoprecipitation PCR		
SCR1-4 Fw	GTCCTGGGCAGAGCTGTCT	Ref. 16
SCR1-4 Rev	AAGGTGGAGCCCCTAAGGA	Ref. 16
tDNA Ileu Fw	GCTCGTGTAGCTCAGTG	Ref. 16
tDNA Ileu Rev	TGCTCGAGGTGGGGTTT	Ref. 16
GAL1 Fw	AAAGAAACTTGCACCGGAAA	Ref. 16
GAL1 Rev	GGCCCATATTCGCTTTAACA	Ref. 16
18S Fw	CTCCGGAATCGAACCCTTAT	Ref. 16
18S Rev	TCGACCCTTTGGAAGAGATG	Ref. 16

[(1)] Dakshinamurthy, A., Nyswaner, K. M., Farabaugh, P. J. & Garfinkel, D. J. BUD22 affects Ty1 retrotransposition and ribosome biogenesis in *Saccharomyces cerevisiae*. *Genetics* **185**, 1193–1205 (2010).

Details on strain and plasmid constructs and mutagenesis will be provided on requests.

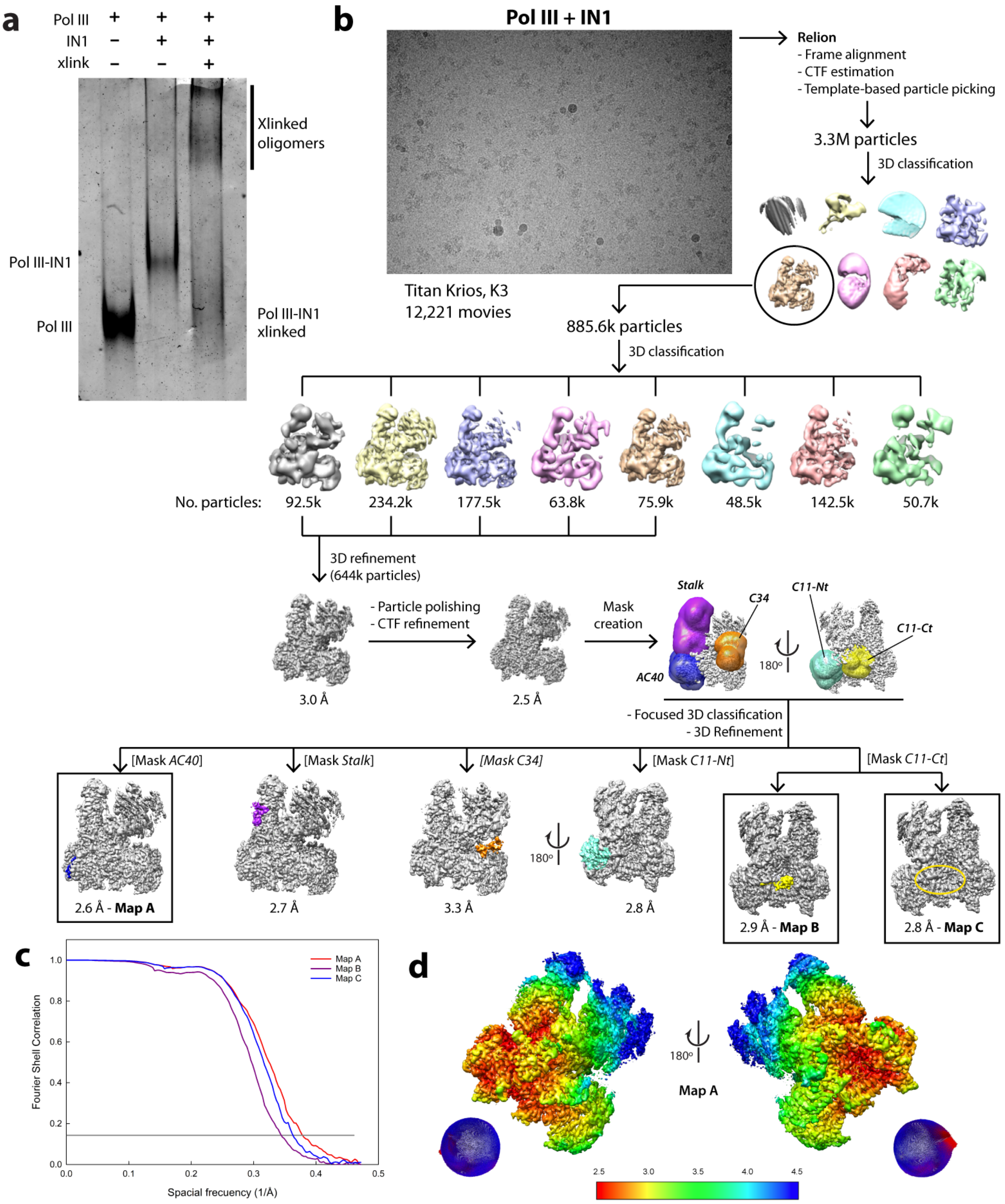

Supplementary Fig. 1. Cryo-EM of the Pol III bound to IN1. (a) Tris-glycine gel showing differential mobility of free Pol III, Pol III bound to IN1, and the latter after crosslinking (xlink). The monomeric species of the crosslinked Pol III bound to IN1 migrates faster than the non-crosslinked sample due to loss of positive charges from crosslinked lysine residues. Higher-order oligomers are discarded during cryo-EM analysis. (b) Processing pipeline of the Pol III bound to IN1 dataset including a representative micrograph. Note that 2D classification was skipped and initial selection of good particles was performed using 3D classification exclusively. (c) FSC curves of maps A, B and C showing a final average resolution of 2.6, 2.9 and 2.8 Å (FSC = 0.143). (d) Local resolution estimation of map A and corresponding angular distribution plot. The two views are related by a 180º rotation around a vertical axis.

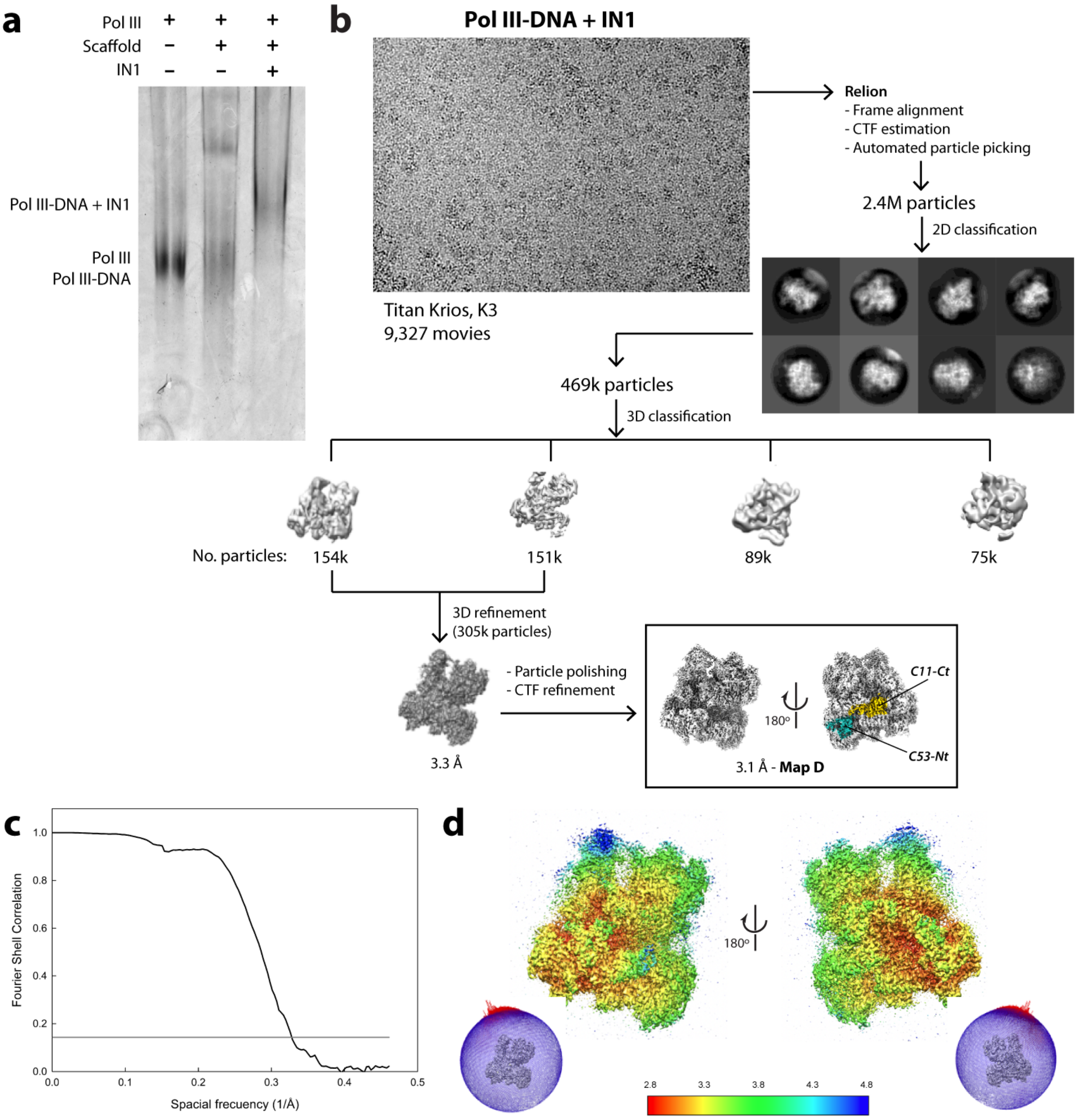

Supplementary Fig. 2. Cryo-EM processing strategy of the complex between Pol III-DNA bound to IN1. (a) 4% Tris-glycine gel showing differential mobility of free Pol III, Pol III-DNA, and the latter after incubation with IN1. (b) Processing pipeline of the Pol III-DNA + IN1 dataset including a representative micrograph. Particle classification with a mask around C11-Ct (identical to that shown in Figure S1A) showed that 294k out of 304k particles present clear density for C11-Ct in the cleft, whereas two other classes with only 6k and 5k particles yield non-interpretable maps. (c) FSC curve of the map derived from the best group of particles, showing a final average resolution of 3.1 Å (FSC = 0.143). (d) Local resolution estimation of the map and corresponding angular distribution plot. The two views are related by a 180º rotation around a vertical axis.

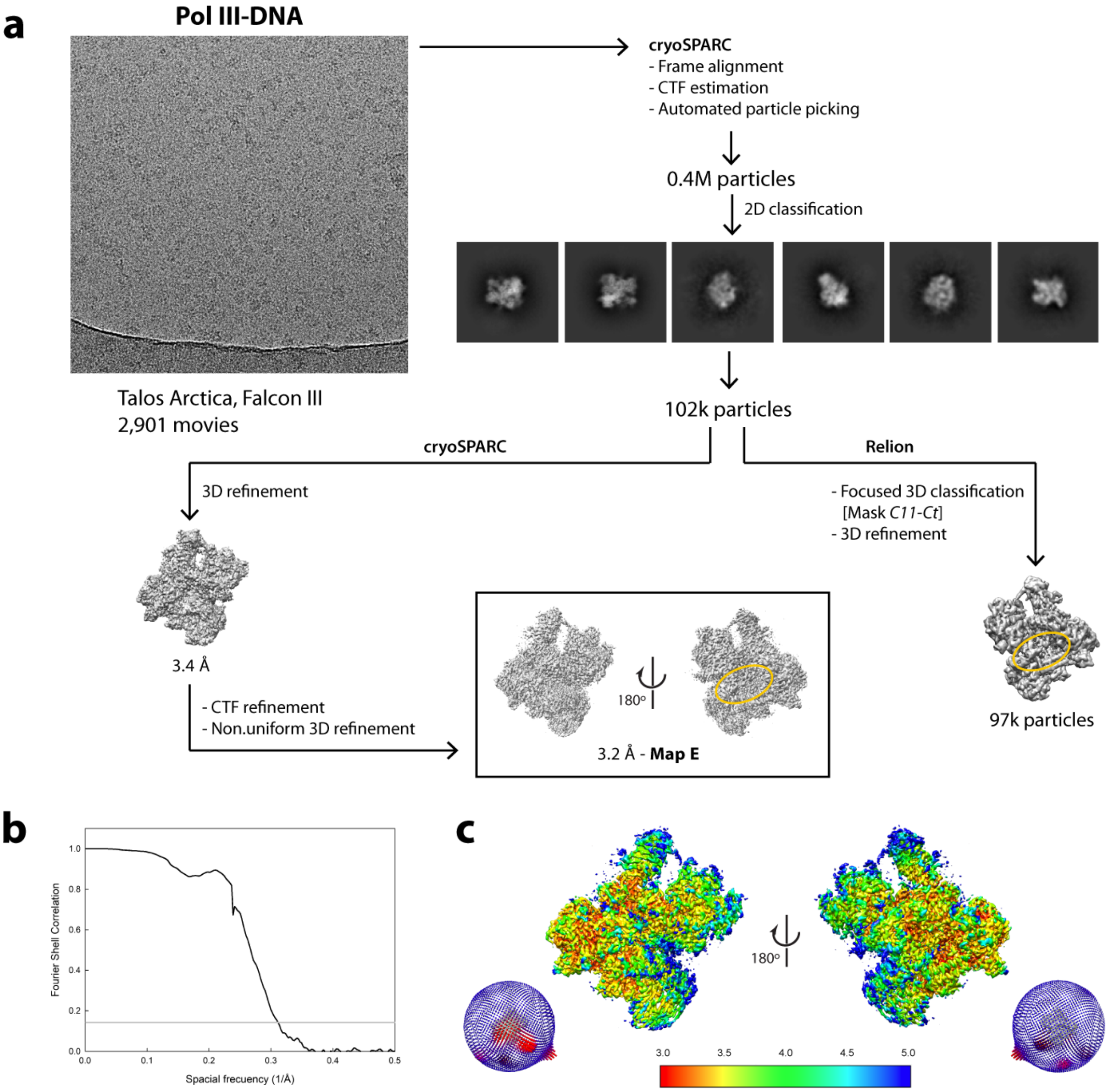

Supplementary Fig. 3. Cryo-EM processing strategy of the Pol III-DNA. (a) Processing pipeline of the Pol III EC-IN1 dataset including a representative micrograph. Particle classification with a mask around C11-Ct (identical to that shown in Figure S1A) showed that 97k out of 102k particles have no density for C11-Ct in the cleft, whereas two other classes with only 3k and 2k particles yield non-interpretable maps. (b) FSC curve of the map, showing a final average resolution of 3.2 Å (FSC = 0.143). (c) Local resolution estimation of the map and corresponding angular distribution plot. The two views are related by a 180º rotation around a vertical axis.

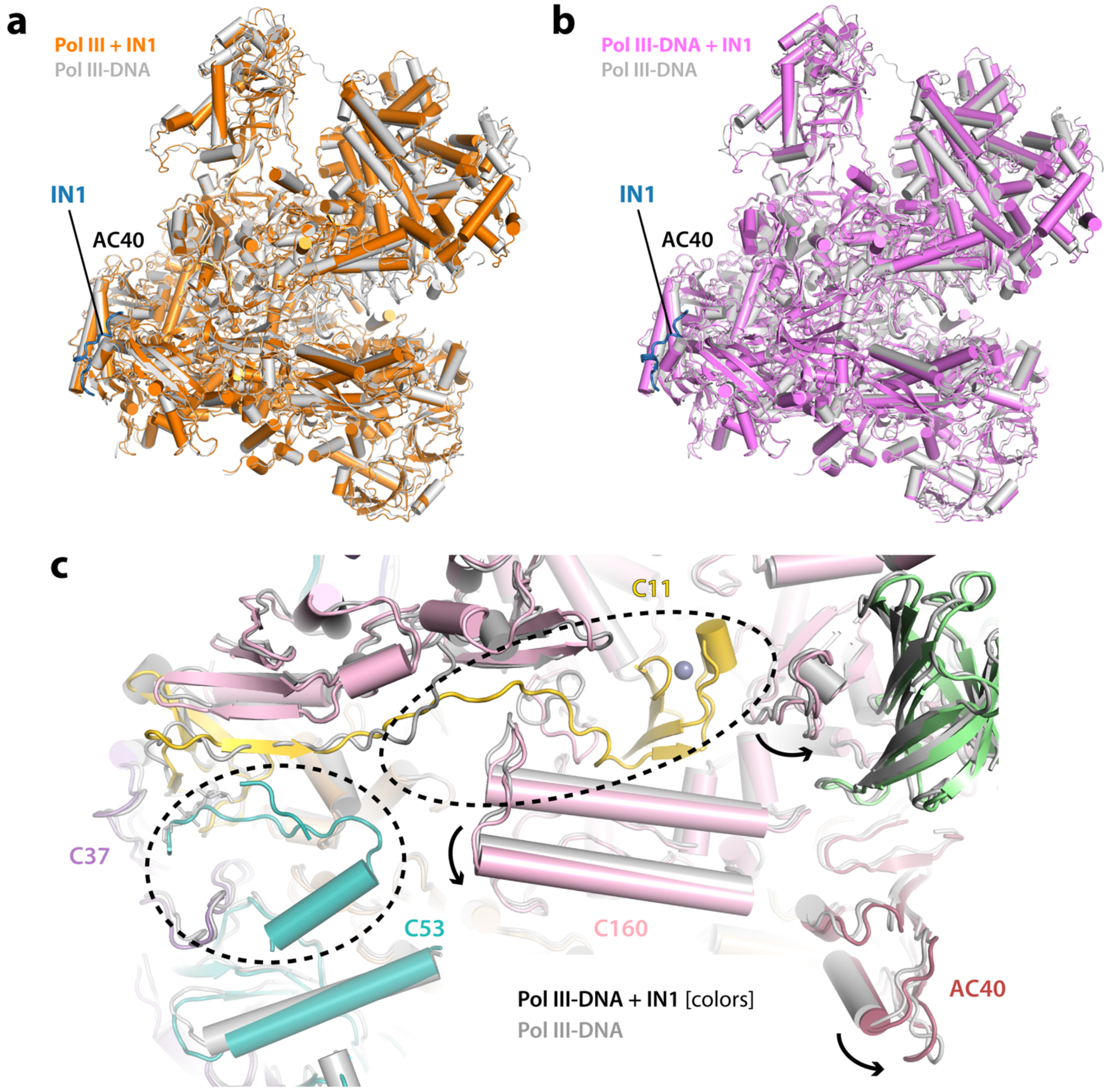

Supplementary Fig. 4. Structural comparison of Pol III bound to IN1 and/or nucleic acids. (a) Superposition of the Pol III bound to IN1 structure (orange) and that of Pol III-DNA (grey), with root-mean-square-deviation (RMSD) of 0.9 Å over 31,993 atoms. (b) Structural superposition of Pol III-DNA bound to IN1 (magenta) and lacking IN1 (grey) with RMSD of 1.2 Å over 35,972 atoms. (c) Close-up view of the superposition shown in panel 'b' after a 180º rotation along a vertical axis. Dotted ovals indicate Pol III regions that are only ordered in the presence of IN1. Arrows indicate Pol III changes upon IN1 binding.

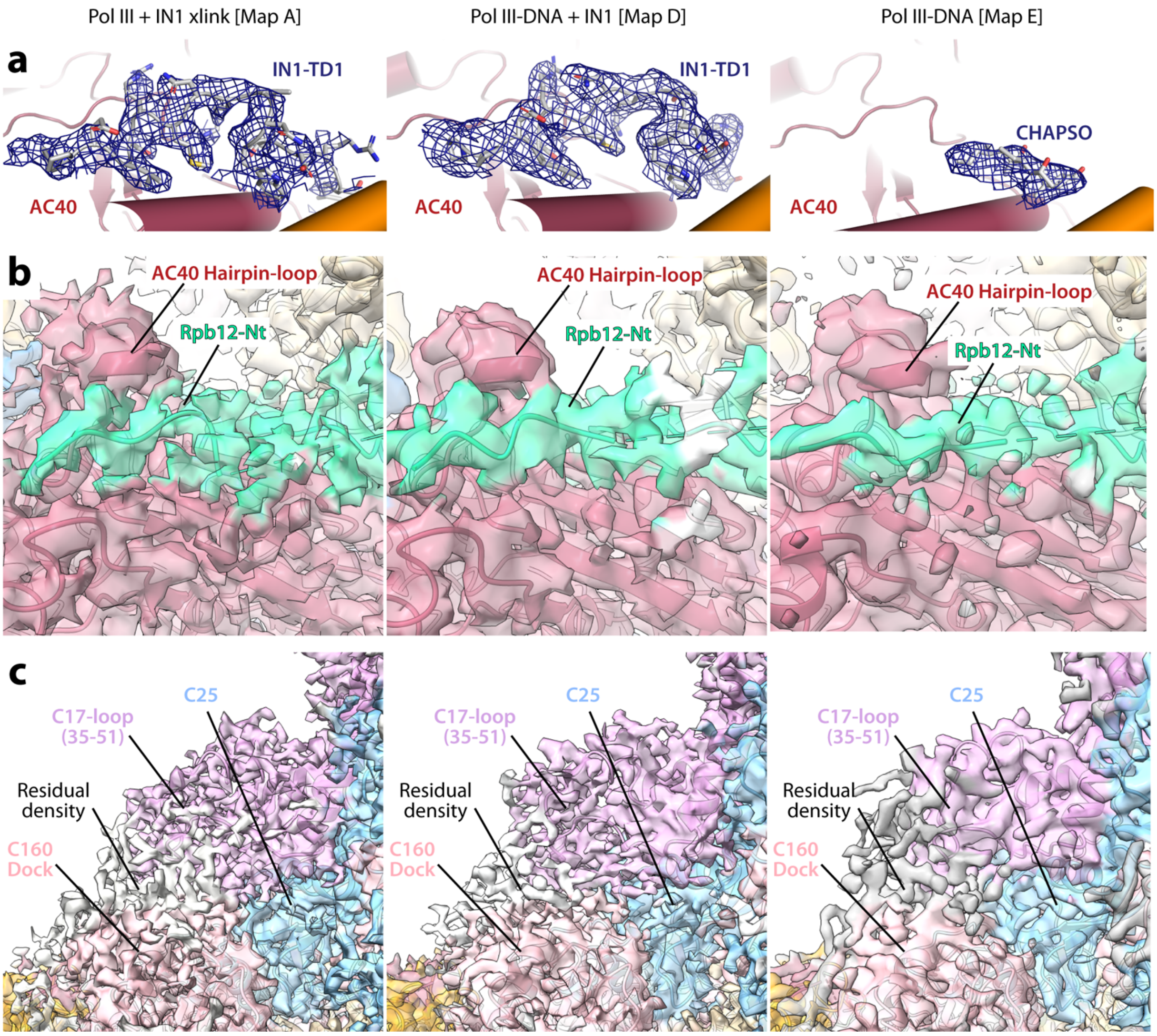

Supplementary Fig. 5. Additional densities in our cryo-EM maps. (a) Pieces of density (dark blue mesh) with corresponding models (grey) at a surface crevice of subunit AC40 (red), as observed in maps of Pol III complexed to IN1 (left), Pol III bound to nucleic acids and IN1 (middle) and Pol III bound to only nucleic acids (right). (b) Elongated density next to AC40 that we tentatively attribute to Rpb12-Nt, as observed in maps of Pol III complexed to IN1 (left), Pol III bound to nucleic acids and IN1 (middle) and Pol III bound to only nucleic acids (right). (c) Residual density (grey) next to stalk subunits (C17, pink; C25, cyan) in maps of Pol III complexed to IN1 (left), Pol III bound to nucleic acids and IN1 (middle) and Pol III bound to only nucleic acids (right).

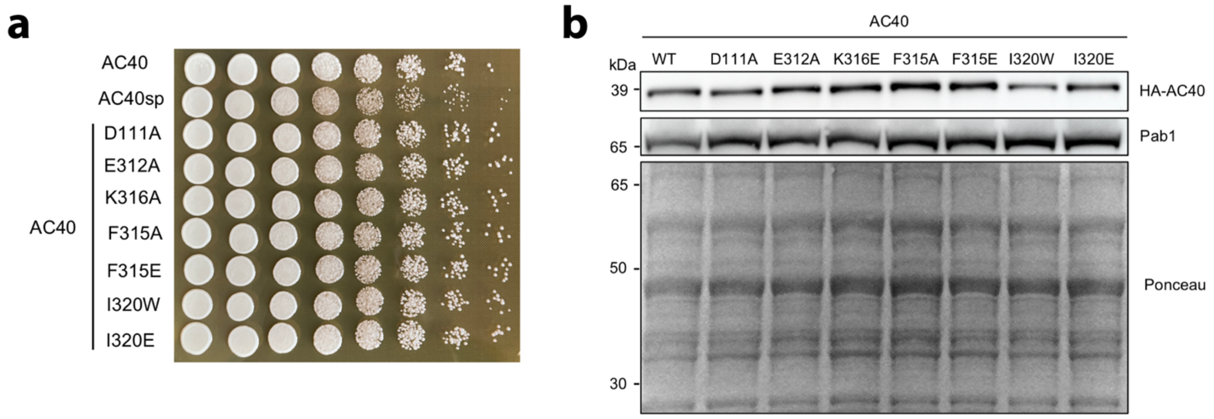

c

Strain genotype	p*GAL1*-Ty1-*his3AI* retrotransposition ± SD ($x10^{-3}$)	p-value (Welch t-test)
AC40	6.03 ± 0.85	-
AC40sp	3.26 ± 0.53	0.0134 (*)
AC40 D111A	3.40 ± 0.89	0.0208 (*)
AC40 E312A	3.09 ± 0.13	0.0246 (*)
AC40 K316A	3.74 ± 0.73	0.0250 (*)
AC40 F315A	6.58 ± 1.20	0.5589 (ns)
AC40 F315E	3.55 ± 0.83	0.0227 (*)
AC40 I320W	3.64 ± 0.76	0.0226 (*)
AC40 I320E	4.06 ± 0.92	0.0528 (ns)

Supplementary Fig. 6. Control experiments related to AC40 mutant analyses. (a) Growth comparison of WT and mutant *RPC40* strains. One OD_{600} of overnight cell culture was used to make 5-fold serial dilutions, starting from 10^0. Cells were dropped on YPD plates and incubated 48h at 30 °C. (b) Expression of HA-AC40 mutant proteins. Whole cell extracts were prepared from 10 OD_{600} of the indicated strain cultures by TCA precipitation, then analyzed by western blotting using monoclonal antibodies anti-HA 12CA5 (Roche; 1:1000), revealing HA-AC40 WT and mutant proteins (top panel). Pab1 serves as a loading control, using monoclonal antibodies anti-Pab1 (Abcam; 1:1000). Total proteins were detected with the reversible Ponceau staining method (bottom panel) and molecular weights are indicated (kDa). (c) Retrotransposition frequency of p*GAL1*-Ty1-*his3AI* in WT and mutant *RPC40* strains. Values are mean ± SD, $n = 3$ experiments, each performed with four independent colonies. *p*-values $*p < 0.05$; ns, not significant. Two-sided Welch's t-test with comparison to the WT strain.

a

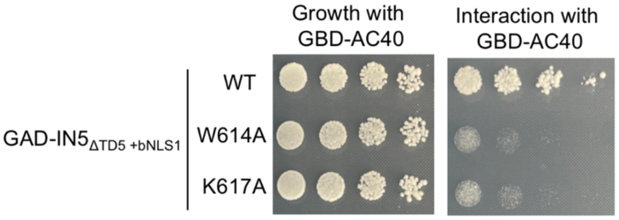

b

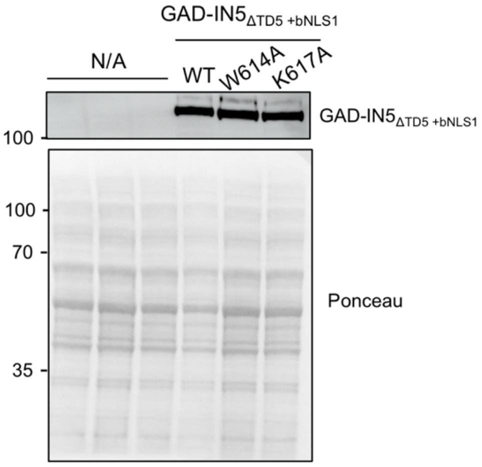

c

IN1 genotype	p*GAL1*-Ty1-*his3AI* retrotransposition ± SD (x10^{-3})	p-value (Welch t-test)
**WT**	5.25 ± 0.81	-
**W614A**	4.23 ± 0.70	0.176432 (ns)

Supplementary Fig. 7. Characterization of IN1 W614A mutant. (a) Two-hybrid interaction between GBD-AC40 and WT or mutant GAD-IN5$_{\Delta TD5+bNLS1}$ in strain PJ69-4A, which contains the *HIS3* reporter to detect positive interactions (Ref. 58). GAD-IN5$_{\Delta TD5+bNLS1}$ harbors the integrase sequence of Ty1 that interacts with AC40 in the integrase sequence of Ty5, in place of the targeting domain of Ty5, and is good indicator of interaction with AC40 (Ref. 16). Cultures of transformants were grown overnight at 30 °C in synthetic complete medium lacking leucine and tryptophan (SC-LEU-TRP) to maintain plasmid selection. 5-fold serial dilutions of aliquots of one OD_{600}, washed in 1 ml of H_20, were plated on SC-LEU-TRP (growth control) or SC-LEU-TRP-HIS (interaction), starting from 10^0. Plates were incubated 2 days at 30 °C. The assay is a representative example of at least two biological replicates. The K617A mutant was used as control for loss of interaction between IN1 and AC40 (Ref. 16). (b) Expression of WT and mutant GAD-IN5$_{\Delta TD5+bNLS1}$ proteins. Whole cell extracts of the indicated strains analyzed by western blotting using monoclonal anti-GAD antibodies (Santa Cruz Biotechnology; 1:1000) (top panel). Total proteins were detected with the reversible Ponceau staining method (bottom panel) and molecular weights are indicated (kDa). N/A are non-relevant samples in this experiment. (c) Retrotransposition frequency of WT and W614A p*GAL1*-Ty1-*his3AI* mutant in a *spt3-101 rad52*Δ strain (LV174) to avoid both trans-complementation of the mutant IN1 by endogenous WT IN1 and Rad52-dependent recombination events (Ref. 16). Values are mean ± SD, n = 3 experiments, each performed with four independent colonies. p-values ns, not significant. Two-sided Welch's t-test with comparison to the WT p*GAL1*-Ty1his3AI.

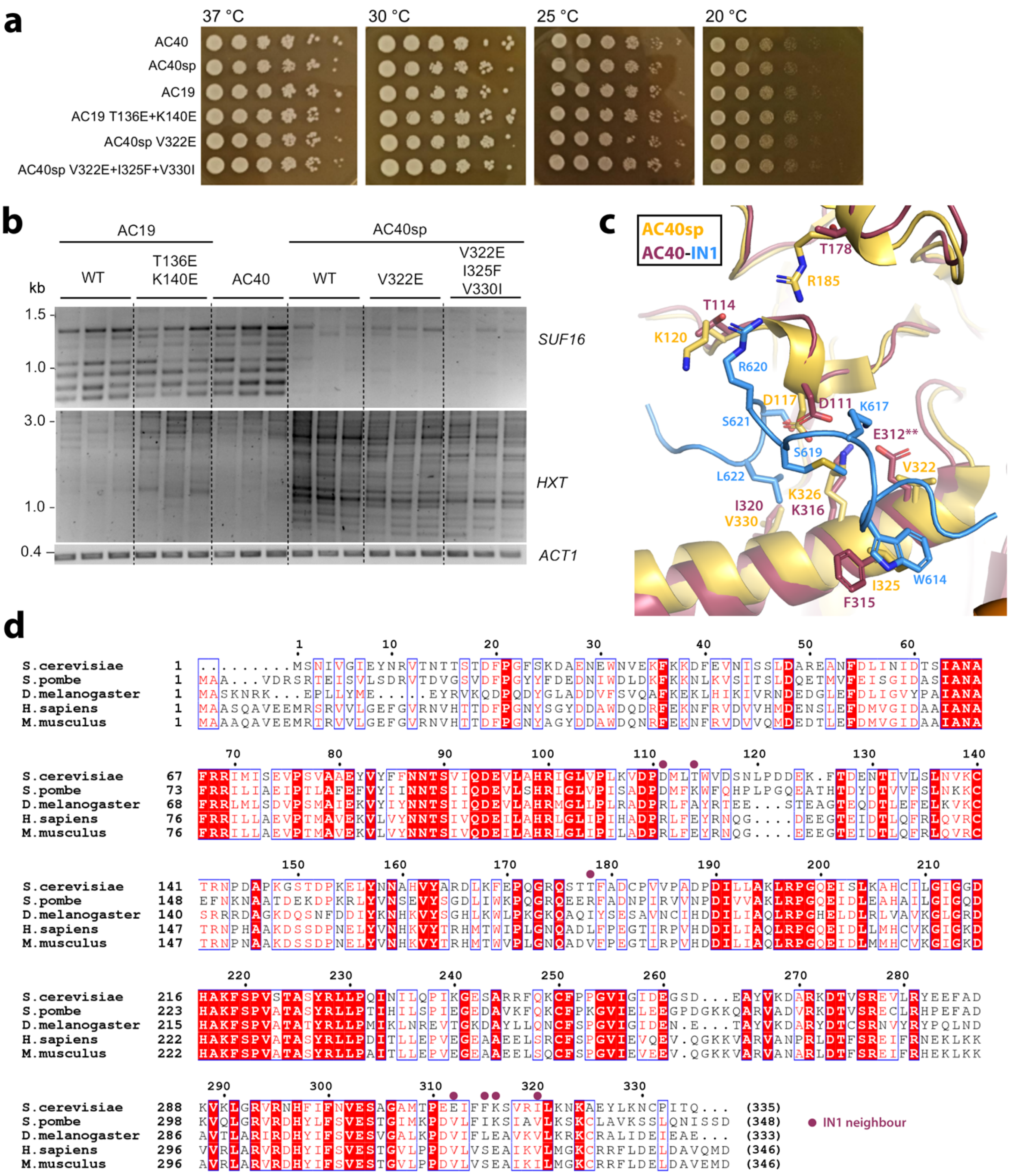

Supplementary Fig. 8. Mutational analysis of subunits AC19 and *S. pombe* AC40. (a) Growth comparison of WT and *RPC19* and *RPC40sp* mutant strains. One OD_{600} of overnight cell culture was used to make 5-fold serial dilutions, starting from 10^0. Cells were dropped on YPD plates and incubated 48h at the indicated temperatures. (b) Detection of endogenous Ty1 insertions upstream of the *SUF16* Pol III-transcribed gene and the *HXT* subtelomeric genes (*HXT13*, *HXT15*, *HXT16* and *HXT17*) by PCR. Endogenous Ty1 retrotransposition was induced in cells growing at 20 °C during 3 days in YPD media. Total genomic DNA was extracted from three independent cultures. *ACT1* is genomic DNA quality control. (c) Structural comparison between *S. cerevisiae* AC40 from Pol III bound to IN1 (dark red) and *S. pombe* AC40 (AC40sp) from available *S. pombe* Pol I coordinates (PDB 7AOE, yellow). TD1 is shown in blue. (d) Alignment of AC40 subunits from different organisms, where a dark purple circle indicates residues in direct contact with IN1 that have been mutated in this study.

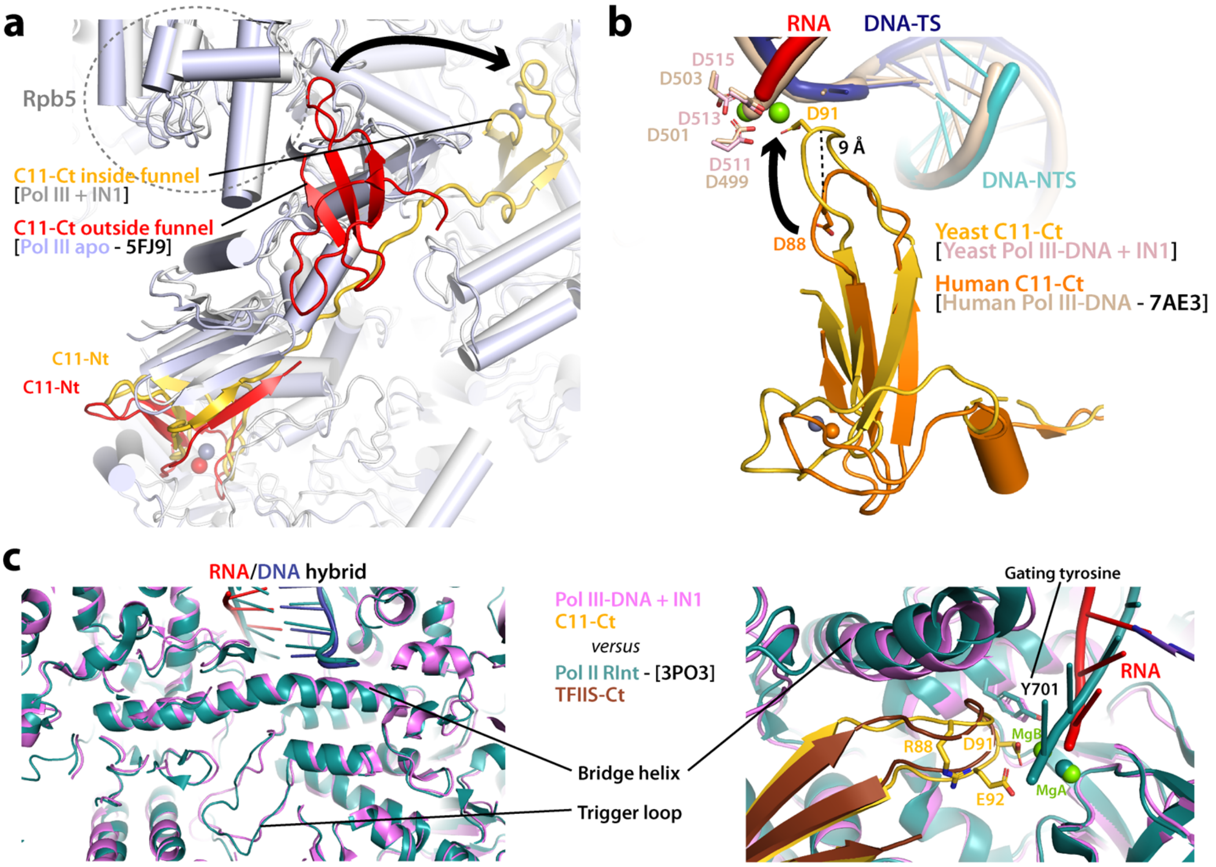

Supplementary Fig. 9. Structural comparisons around subunit C11. (a) Structural comparison of Pol III bound to IN1 (grey) with free Pol III (PDB 5FJ9, light blue). C11 in these structures is in yellow and red, respectively. (b) Comparison of the yeast Pol III bound to IN1 structure with human Pol III elongation complex (PDB 7AE3, light brown). Nucleic acids in the Pol III-DNA + IN1 structure (RNA/DNA hybrid modelled from poor density, especially for RNA) are cyan, blue and red for the non-template strand (NTS) of DNA, template strand (TS) of DNA and RNA, respectively. (c) Structural comparison of the yeast Pol III bound to IN1 structure (magenta) with Pol II-TFIIS in the rescue intermediate complex (RInt; PDB 3PO3, teal), with close-up views around the bridge helix (left panel) and C11-Ct (right panel).

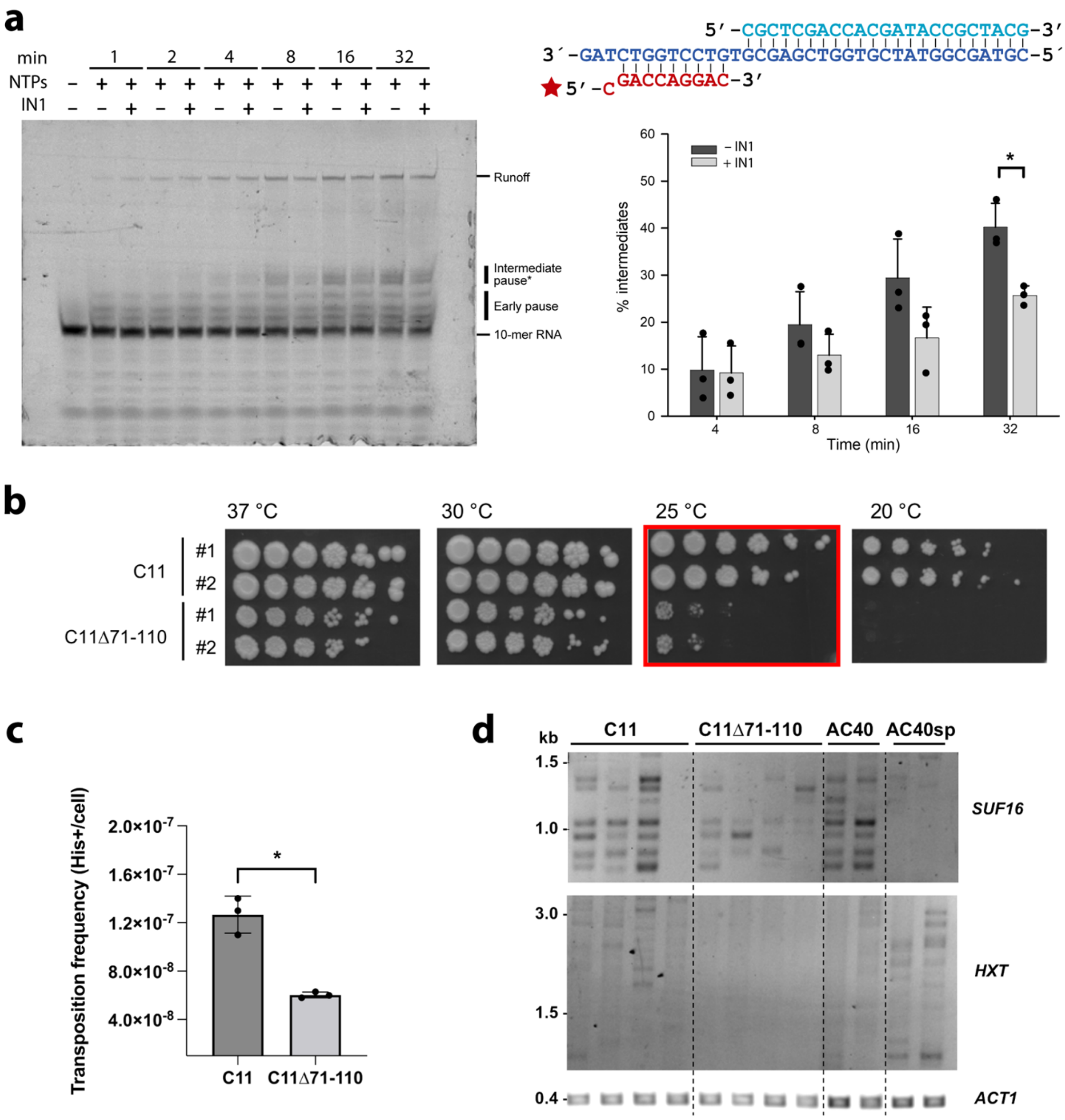

Supplementary Fig. 10. Characterization of C11-Ct role in the presence of IN1. (a) RNA extension assay of Pol III (100 nM) in the absence (-) or in the presence of IN1 (200 nM) at different time points. The nucleic acid scaffold is on the top-right, with RNA in red and DNA in cyan and blue. A red star indicates a fluorescent label on the RNA 5' end. Quantification of the intermediate pause bands (see gel on the left) from 3 independent experiments, with mean ± SD. *p*-values $^*p < 0.05$. Two-sided Welch's t-test. (b) Growth comparison of WT and *rpc11Δ71-110* mutant strains. One OD_{600} of overnight cell culture was used to make 5-fold serial dilutions, starting from 10^0. Cells were dropped on YPD plates and incubated 72h at the indicated temperatures. A red square indicates the temperature for experiments in panel 'c'. (c) Retrotransposition frequency of a chromosomal Ty1-*his3AI* reporter in WT and mutant *rpc11Δ71-110* strains. Values are mean ± SD, $n = 3$ experiments, each performed with four independent colonies. *p*-values $^*p < 0.05$. Two-sided Welch's t-test. (d) Detection of endogenous Ty1 insertions upstream of the *SUF16* Pol III-transcribed gene and the *HXT* subtelomeric genes (*HXT13*, *HXT15*, *HXT16* and *HXT17*) by PCR. Endogenous Ty1 retrotransposition was induced in cells growing at 25°C during 3 days in YPD media. Total genomic DNA was extracted from three independent cultures. *ACT1* is genomic DNA quality control. *Rcp40Δ* strains transformed by a plasmid expressing AC40 or AC40sp are used as controls.

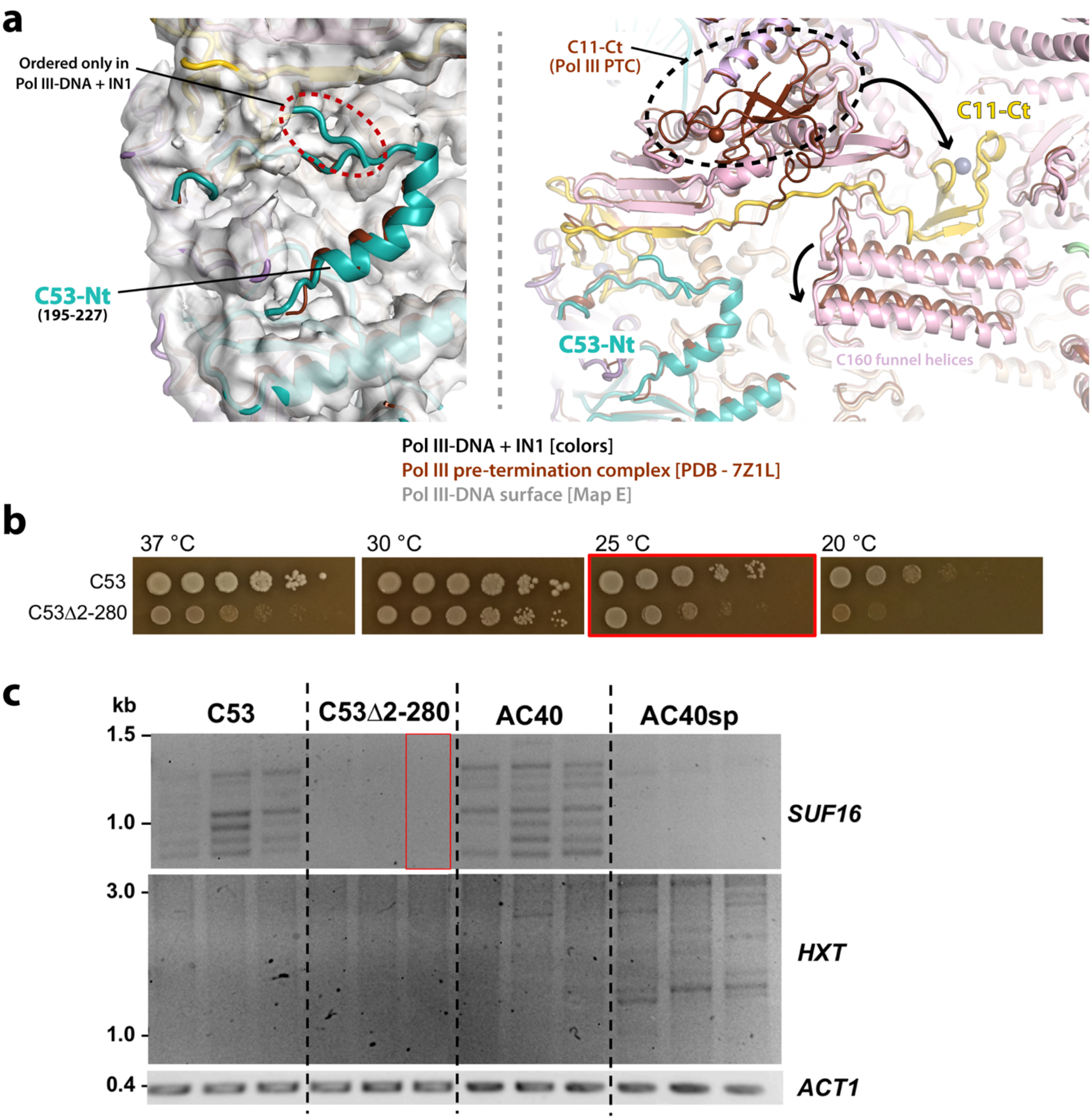

Supplementary Fig. 11. Characterization of C53-Nt role in the presence of IN1. (a) View around C53-Nt in the structure of Pol III-DNA bound to IN1 (color ribbon), fitted in the Pol III-DNA map lacking IN1 (grey surface), and superposed with Pol III pre-termination complex (PTC) structure (brown). (b) Growth comparison of the following strains: WT, mutant *rpc53Δ2-280* and mutant *rpc53Δ2-280 rpc40Δ* transformed by a plasmid expressing AC40sc or AC40sp. One OD_{600} of overnight cell culture was used to make 5-fold serial dilutions, starting from 10^0. Cells were dropped on YPD plates and incubated 48h at the indicated temperatures. A red square indicates the temperature for experiments in panel 'b'. (c) Detection of endogenous Ty1 insertions upstream of the *SUF16* Pol III-transcribed gene and the *HXT* subtelomeric genes (*HXT13*, *HXT15*, *HXT16* and *HXT17*) by PCR. Endogenous Ty1 retrotransposition was induced by growing the indicated strains at 25 °C during 3 days in YPD media. Total genomic DNA was extracted from three independent cultures. *ACT1* is genomic DNA quality control. A red box indicates an empty lane due to lack of PCR amplification.

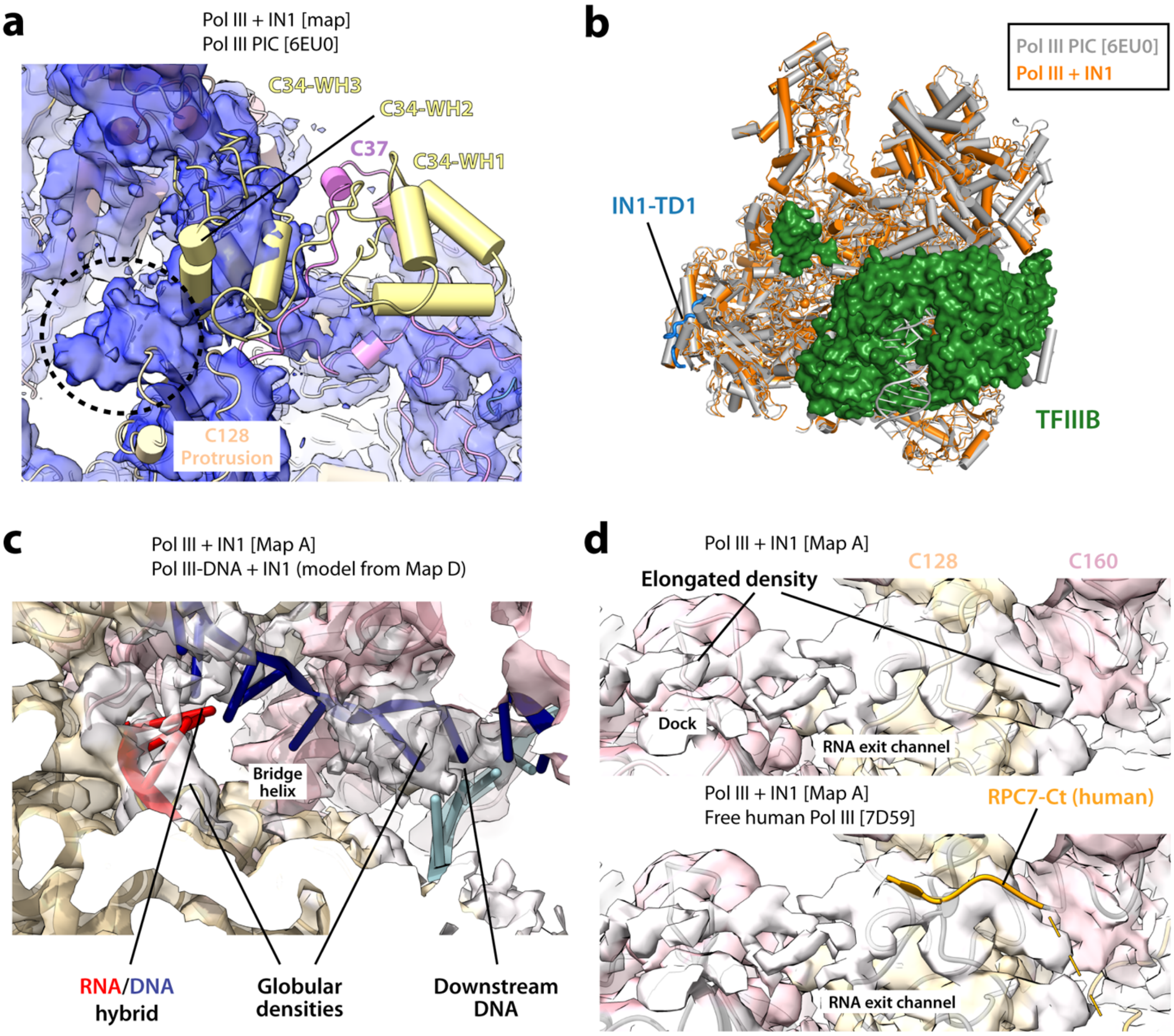

Supplementary Fig. 12. Additional densities in the Pol III bound to IN1 lacking nucleic acids. (a) Schematic representation of C34 and view of the Pol III-IN1 map around C34, where the structure of Pol III in the preinitiation complex (PIC, PDB 6EU0, left) or complexed to Maf1 (PDB 6TUT, right) has been fitted. (b) View of the Pol III-IN1 map obtained from particle classification using a mask around C34, where the structure of Pol III PIC (PDB 6EU0) has been fitted. A dotted circle indicates the presence of an additional piece of density apart from that attributed to C34-WH2. (c) Close-up view of the cleft in the map of Pol III + IN1 with fitted model of Pol III-DNA + IN1 showing globular densities in the absence of nucleic acids that partly overlap with downstream DNA and the RNA/DNA hybrid. DNA strands are in dark (template) and light (non-template) blue while RNA is in red. (d) View of the RNA exit channel in the Pol III bound to IN1 map with its corresponding model (upper panel) or the structure of free human Pol III (PDB 7D59; lower panel).

Source Data for Supplementary Fig. 1

Panel A

Experiment 08/09/2020 (S. H.)

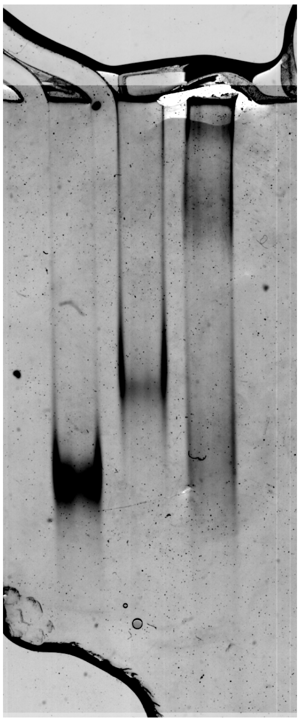

Source Data for Supplementary Fig. 2

Panel A

Experiment 15/12/2020 (S. H.)

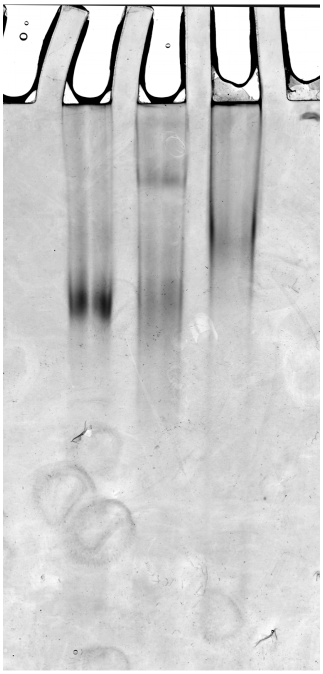

Source Data for Supplementary Fig. 6

Panel B
Experiment 28/06/2022 (N. P. + A. A.-L.)

Gel order

	HA-AC40
Lane 1	D111A
Lane 2	E312A
Lane 3	K316E
Lane 4	F315A
Lane 5	F315E
Lane 6	I320W
Lane 7	I320E

Size marker kDa (top to bottom)

	Page Ruler
185	Blue
115	Blue
80	Blue
65	Orange
50	Blue
30	Blue
25	orange

Ponceau

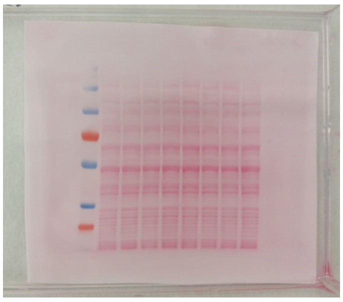

Anti-Pab1: (~ 64kDa)

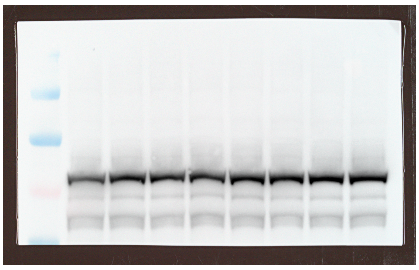

Anti-HA (AC40-HA ~ 40kDa)

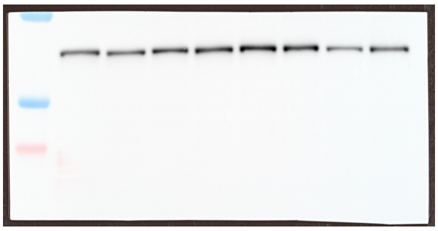

Panel C
Experiments 25/08/2021 (A. A.-L.)

**Souches**	**Median n=1**	**Madian n=2**	**Median n=3**	**Mean of medians**	**Standard deviation**
**AC40sc**	5.27E-03	5.87E-03	6.95E-03	**6.03E-03**	8.52E-04
**AC40sp**	2.69E-03	3.34E-03	3.75E-03	**3.26E-03**	5.34E-04
**AC40sc D111A**	2.49E-03	3.44E-03	4.27E-03	**3.40E-03**	8.89E-04
**AC40sc E312A**	3.17E-03	2.94E-03	3.16E-03	**3.09E-03**	1.29E-04
**AC40sc K316A**	3.06E-03	4.52E-03	3.64E-03	**3.74E-03**	7.35E-04
**AC40sc F315A**	6.85E-03	5.26E-03	7.62E-03	**6.58E-03**	1.20E-03
**AC40sc F315E**	3.87E-03	4.18E-03	2.61E-03	**3.55E-03**	8.34E-04
**AC40sc I320W**	3.14E-03	3.26E-03	4.51E-03	**3.64E-03**	7.61E-04
**AC40sc I320E**	4.82E-03	3.04E-03	4.32E-03	**4.06E-03**	9.16E-04

Medians of 4 independent cultures
Statistic n=3 experiments
Welch's t-test performed in R

**Strain genotype**	**Ty1-his3AI retromobility ± SD (x10-3)**	***p*-value (Welch t-test)**	
**AC40sc**	6.03 ± 0.85	N/A	
**AC40sp**	3.26 ± 0.53	0.0134	*
**AC40sc D111A**	3.40 ± 0.89	0.0208	*
**AC40sc E312A**	3.09 ± 0.13	0.0246	*
**AC40sc K316A**	3.74 ± 0.73	0.0250	*
**AC40sc F315A**	6.58 ± 1.20	0.5589	ns
**AC40sc F315E**	3.55 ± 0.83	0.0227	*
**AC40sc I320W**	3.64 ± 0.76	0.0226	*
**AC40sc I320E**	4.06 ± 0.92	0.0528	ns

*$p < 0.05$; **$p < 0.01$; ***$p < 0.001$

Source Data for Supplementary Fig. 7

Panel A

Experiment 30/09/2021 (A. A.-L.)

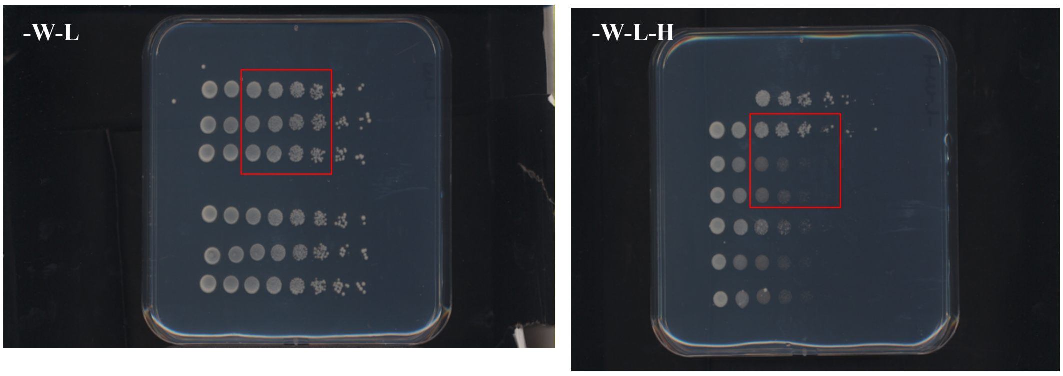

Panel B

Experiment 07/09/2021 (N. P. + A. A.-L.)

Size marker kDa (top to bottom). Ponceau anti-GAD (GAD-IN5-ΔTD5+bNLS1)

	Page Ruler
115	Blue
80	Blue
65	Orange
50	Blue
40	Blue
30	Blue
25	Blue

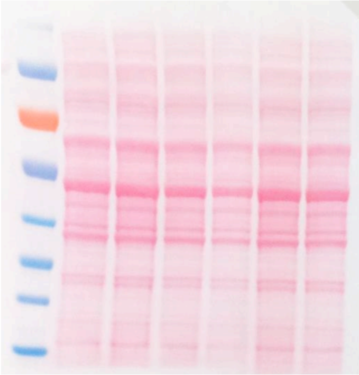

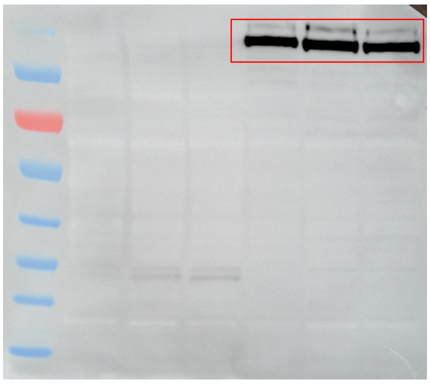

Panel C

Experiments 26/07/2021-02/08/2021 (A. A.-L.)

**pGAL-Ty1-his3AI**	**Median n=1**	**Median n=2**	**Median n=3**	**Mean**	**Standard deviation**	**Ty1-his3AI retromobility ± SD (x10-3)**	***p*-value (Welch t-test)**
**WT**	6.18E-03	4.92E-03	4.65E-03	**5.25E-03**	8.17E-04	5.25 ± 0.81	N/A
**W614A**	4.45E-03	4.79E-03	3.44E-03	**4.23E-03**	7.02E-04	4.23 ± 0.70	0.176432 (ns)

Medians of 4 independent cultures

Statistic n=3 experiments

Welch's t-test performed in R

Source Data for Supplementary Fig. 8

Panel A
Experiment 23/11/2022 (B. C.)
48 hrs

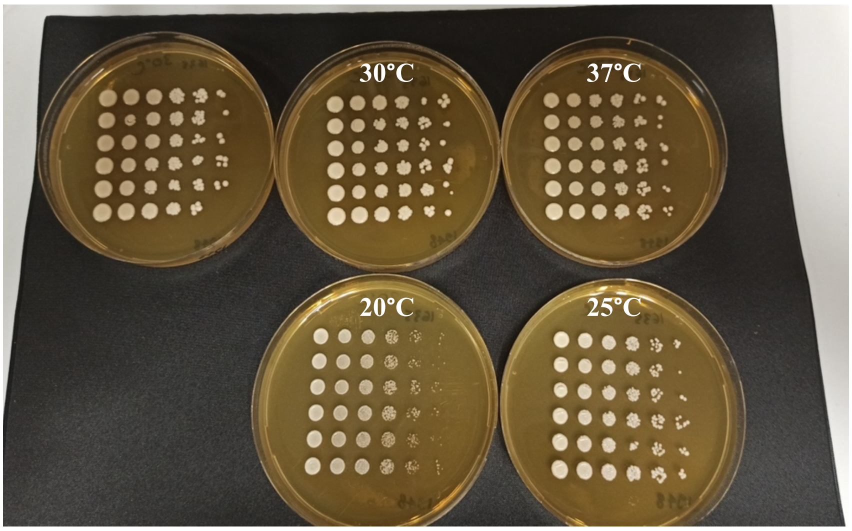

Panel B
Experiment 30/11/2022 (B. C.)
ACT1 PCR:

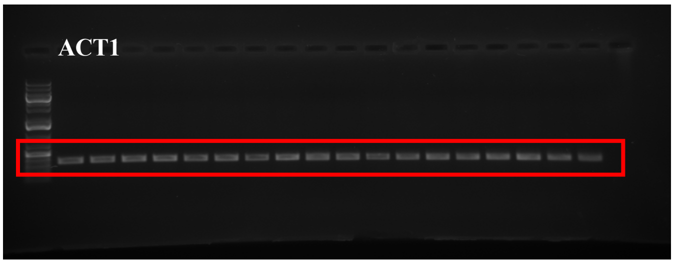

Experiment 29/11/2022 (B. C.)
HXT PCR:

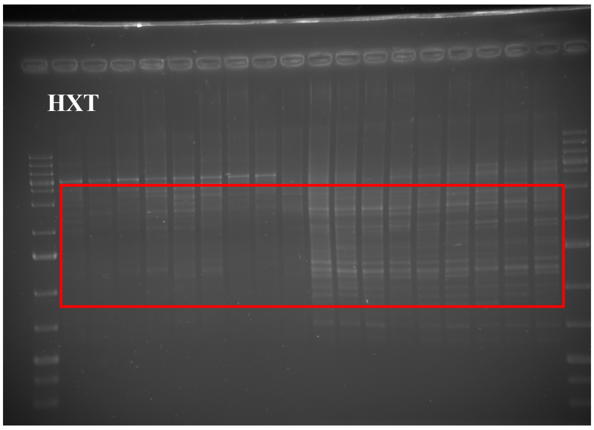

Experiment 25/11/2022 (B. C.)
SUF16 PCR:

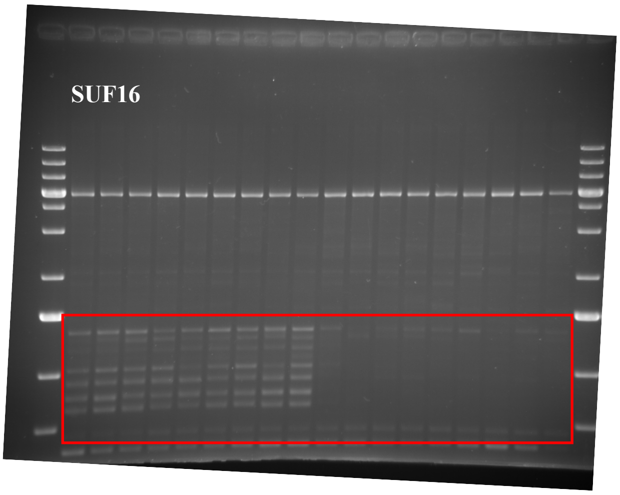

Source Data for Supplementary Fig. 10

Panel A

Experiment 20/12/2021 (S. H.)

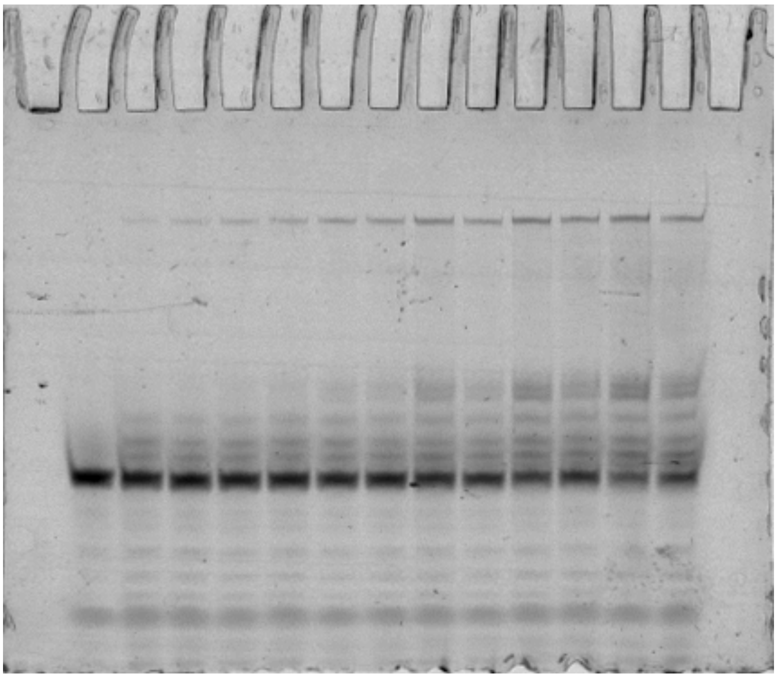

	**% of early intermediates**		
Time (min)	Gel 1	Gel 2	Gel 3
32 +IN1	23.56	27.66	25.82
32 –IN1	37.68	36.88	46.07
16 +IN1	9.19	21.35	19.44
16 –IN1	23.02	26.34	38.79
8 +IN1	9.78	11.14	18.07
8 –IN1	15.49	15.32	27.56
4 +IN1	4.43	7.64	15.54
4 –IN1	3.86	7.90	17.66

	**+ IN1**		**– IN1**	
Time (min)	Mean	stddev	Mean	stdev
4	9.81	7.09	9.21	5.72
8	19.46	7.02	13.00	4.45
16	29.39	8.31	16.66	6.54
32	40.21	5.09	25.68	2.05

Panel B
Experiment 27/10/2022 (B. C.)
48 hrs:

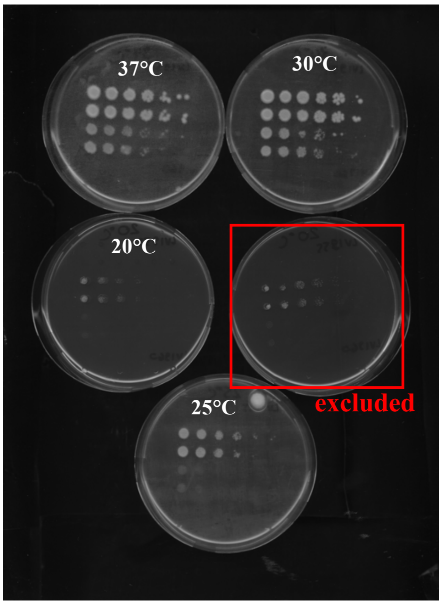

Panel D
PCR SUF16 and HXT: 24/11/2022 (B. C.)

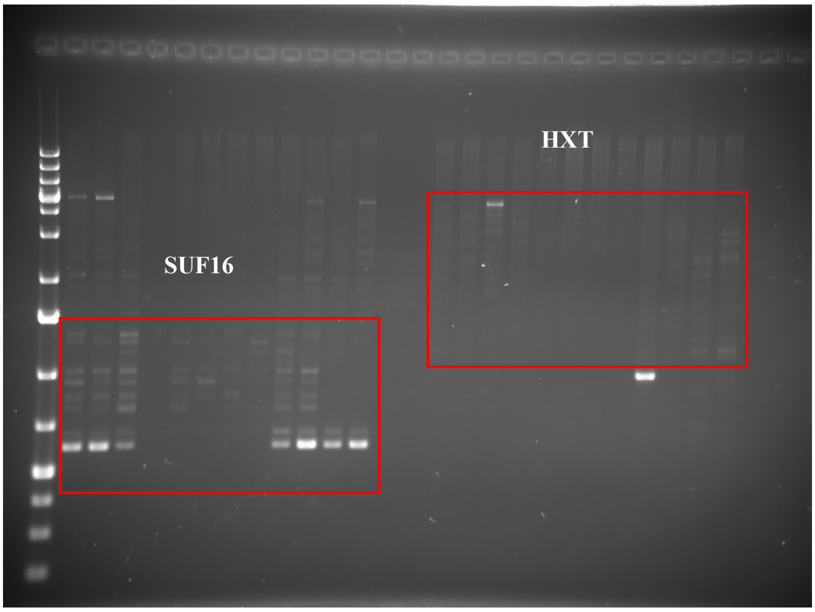

PCR ACT1: 30/11/2022 (B. C.)

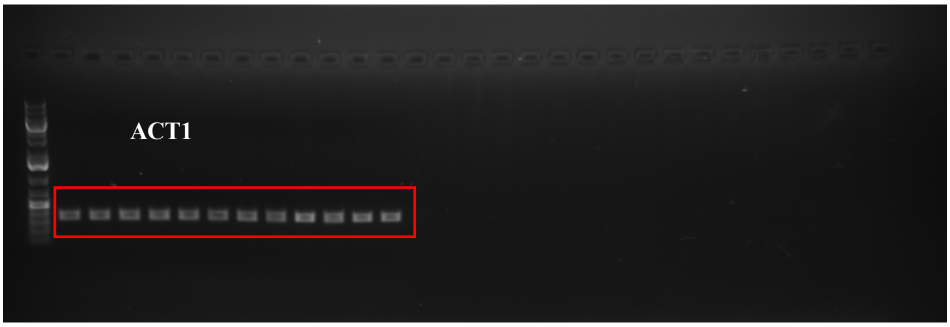

Panel C
Experiments (B. C.)

	07/11/2022		24/11/2022		06/11/2022	
	**Median***	**SD**	**Median***	**SD**	**Median***	**SD**
C11 wt	**1.1E-07**	1.1E-07	**1.3E-07**	1.9E-08	**1.4E-07**	7.5E-08
C11Δ71-110	**5.8E-08**	3.9E-08	**6.0E-08**	4.4E-08	**6.3E-08**	4.4E-09

*Median of 4 independent cultures

Statistics (Prism 9.5.0)

**Welch test**	
Table Analyzed	Data 1 strains LV1955 and LV1959
Column B	C11Δ71-110
vs.	vs.
Column A	C11
Unpaired t test with Welch's correction	
p-value	0.0152
p-value summary	*
Significantly different ($p < 0.05$)?	Yes
One- or two-tailed P value?	Two-tailed
Welch-corrected t, df	t=7.421, df=2.108
How big is the difference?	
Mean of column A	1.267E-07
Mean of column B	6.033E-08
Difference between means (B - A) ± SEM	-6.633e-008 ± 8.938e-009
95% confidence interval	-1.030e-007 to -2.971e-008
R squared (eta squared)	0.9631
F test to compare variances	
F, DFn, Dfd	36.84, 2, 2
p-value	0.0529
p-value summary	ns
Significantly different (P < 0.05)?	No
Data analyzed	
Sample size, column A	3
Sample size, column B	3

Source Data for Supplementary Fig. 11

Panel A

Experiment 23/11/2022 (B. C.)

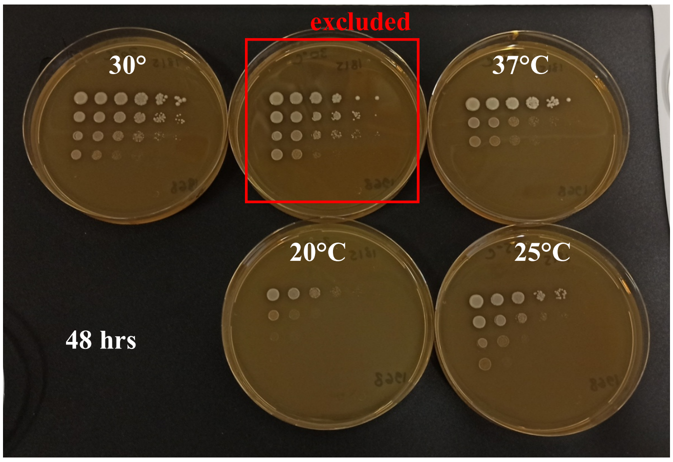

Panel B

Experiment 12/12/2022 (B. C.)

PCR SUF16:

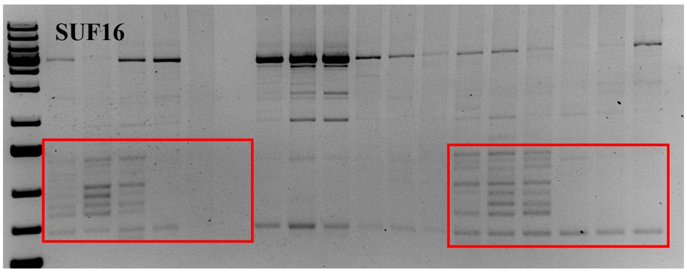

PCR HXT:

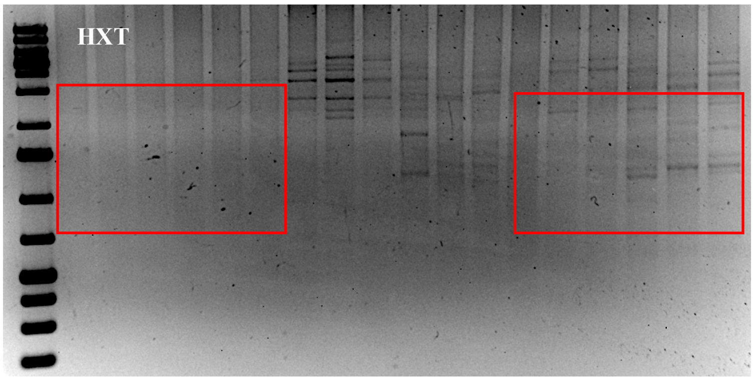

PCR ACT1:

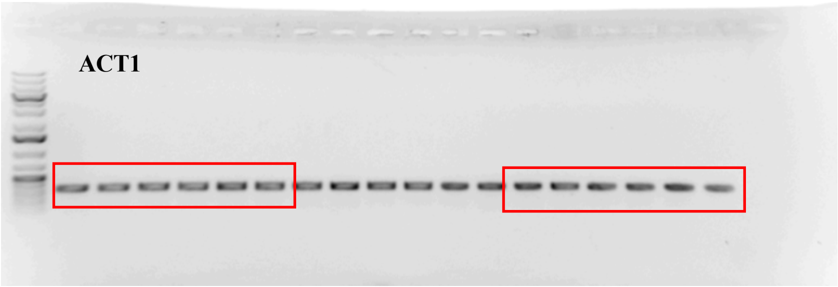

Supplement: Supplementary file 1 — Supplementary Information [file 41467_2023_37109_MOESM1_ESM.pdf]
